# Supplementary material for: Global and national trends, gaps, and opportunities in documenting and monitoring species distributions
Source: PLoS Biol. 2021 Aug 12;19(8):e3001336. doi: 10.1371/journal.pbio.3001336 (PMC8360587; doi:10.1371/journal.pbio.3001336)
Supplement: S1 File — Text A. Methods, Supplementary Text, and Supplementary Acknowledgments to support main text. Fig A. National patterns in data collection, coverage, and sampling effectiveness (2010–2019). (a) Change rates in Steward’s SSII and National SSEI. Dashed lines represent zero slopes. (b, c) Relationship and mismatch between Steward’s SSII and total spatiotemporal records collected nationally (b) and the percentage of expected species nationally recorded (c). (d) Relationship between the percentage of expected species nationally recorded and mean National SSEI. The data underlying this figure may be found in https://mol.org/indicators/coverage and https://github.com/MapofLife/biodiversity-data-gaps. Fig B. National stewardship in data coverage. (a) National and Steward’s SSII over the previous decade (2010–2019). Points are colored by the percent difference between National and Steward’s SSII. Dashed line represents the 1:1 line between variables. (b) Relative stewardship of nations, as estimated by percent difference, over the previous decade. Color scale matches that in panel (a). National boundaries from gadm.org. The data underlying this figure may be found in https://mol.org/indicators/coverage and https://github.com/MapofLife/biodiversity-data-gaps. Fig C. Empirical demonstration of the effects of spatial resolution on the SSII and SSEI. (a, b) Thresholded species distribution model output (Ellis-Soto and colleagues, 2021) rescaled to 3 spatial resolutions (110, 55, and 27.5 km) for 2 hummingbird species, (a) the Glowing puffleg (Eriocnemis vestita) and (b) White-sided hillstar (Oreotrochilus leucopleurus). Grid cells are colored by the number of records collected between 2000–2019. (c) Annual SSII (solid lines) and SSEI (dashed lines) computed at 3 spatial resolutions. (d–i) Comparison of SSII (d–f) and SSEI (g–i) values among spatial resolutions (d, g: 100 vs. 55 km; e, h: 55 vs. 27.5 km; f, i: 110 vs. 27.5 km). Gray shading shows 95% confidence interval. Colored te [file pbio.3001336.s001.pdf]

Supporting Information

Global and national trends, gaps, and opportunities in documenting and monitoring species distributions

Ruth Y. Oliver, Carsten Meyer, Ajay Ranipeta, Kevin Winner, Walter Jetz
Correspondence to: ruth.oliver@yale.edu, walter.jetz@yale.edu

This PDF file includes:
S1 Text A
S1 Figs A-D
S1 Tables A-C

S1 Text: Methods

Species distribution data

Spatial biodiversity data coverage, hereafter referred to as data coverage, can be calculated at any spatial, temporal, or taxonomic resolution of interest. Doing so requires an estimation of expected biodiversity. The spatial resolution of analysis is primarily determined by the data used to estimate a species expected range. That is, higher spatial uncertainty in the species range expectations necessitates coarser resolutions of analysis in order to minimize false presences and thereby not hold nations accountable for collecting data in areas where a species does not occur. We estimated terrestrial vertebrate diversity based composites of single species distribution maps. Previous empirical studies have shown that such expert-based range maps minimize false presences at spatial resolutions of 100-500 km to approximate species' global extents over long time periods (ca. 10-50 years) (1–3). Therefore, we estimated diversity using an equal-area grid with the finest spatial grain appropriate for expert-based species distribution maps (110km x 110km). We demonstrated the potential to estimate species distributions at finer spatial resolutions (55 and 27.5 km) based on output from published species distribution models (4) for two example species (S1 Fig. C). We determined expected diversity for terrestrial birds (N = 9687) (5), mammals (N = 5513) (6), amphibians (N = 6275) (7), and reptiles (N = 9574) (8) using a global equal-area grid with the finest spatial grain appropriate (110 x 110 km at the equator).

For bird species, we restrict our analysis to breeding and resident ranges, excluding migration and wintering habitats. Our analysis therefore expects nations to assess distribution status for any breeding species annually, irrespective of the duration that the species resides within the country.

The grid cells intersecting a species range map were considered to be expected occupied by that species, and thus served as the baseline against which existing records were compared to. Our estimates of expected terrestrial vertebrate diversity are based on static estimates of species ranges and thus does not capture range shifts which may have occurred during our study period (1950-2019) (9–13). Our use of species distributions coarsened to 110 km grid

cells to reduce false presences, may help alleviate this issue, as the magnitude of shifts for terrestrial vertebrates will often fall within this expectation (11). However, we acknowledge that species range shifts may impact even our broad-scale baseline of cross-taxon diversity patterns. Our data coverage metrics may be most strongly affected by recent range shifts driven by climate change (11) as well as land-use change and interacting effects on species ranges (14,15). However, the indices and associated analytical framework we have developed can flexibly support dynamic range expectation information as they become available.

As a representation of digitally accessible and publicly available spatiotemporal biodiversity records, we compiled over one billion occurrence records for terrestrial vertebrates (downloaded April 2020), aggregated by the Global Biodiversity Information Facility (16), of which 454 million were taxonomically and spatially valid and unique. Duplicate records with the same species name, coordinates, and date were removed. To link GBIF-facilitated records to species range maps, we performed taxonomic harmonization based on synonym lists built for this specific purpose. We used scientific names associated with records, which are pre-filtered through GBIF's backbone taxonomy. Records were considered taxonomically valid if the scientific name could be resolved based on custom-built synonym lists which aggregated synonyms to link to accepted names from additional databases. We followed species delimitations for birds from (17), for mammals from (18), for amphibians from (19), and for reptiles from (20). We linked accepted scientific names to potential synonyms and typographical variants compiled from additional data sources (6,7,21–23). We restricted our analysis to unambiguous synonyms to avoid matching records to multiple species. We considered records spatially valid based on their intersection with gridded species expert range maps. Restricting records which occurred within expert expectations may exclude true presences, however doing so eliminates errant records as well as those originating from captive or invasive animals.

Species Status Information Index (SSII)

For a given species, the Species Status Information Index (SSII) captures how well existing data covers the species' expected range. At the species level, the SSII can be computed across the entirety of the species' expected range, ignoring national boundaries, or separately within each nation where it is expected to occur. The global SSII,$I_i = O_i/E_i$, for species *i* across its entire range is the proportion of expected cells, E_i, with records over a given timespan, O_i.

At the national level, for a given taxon with S_c species expected in country *C*, we define the SSII, I_C, as follows, distinguishing two formulations (Fig. 1):

1. **National SSII:** This index measures how well, on average, species are documented in a given nation over a given timespane, in this case per year. The index value *I* for country *C* in a particular year is given by the arithmetic mean among expected species S_c of the proportion of expected cells in country *C*, E_c, with records from that year, O_c (Fig. 1b):
$$I_C = \frac{1}{S_C}\sum_{i=1}^{S_C}\frac{O_{Ci}}{E_{Ci}}.$$
2. **Steward's SSII:** This index adjusts the national coverage based on nations' stewardship of species, upweighting the documentation of species for which a nation has particularly high stewardship. National species stewardship $N_{Ci} = E_{Ci}/E_{.i}$is defined as the proportion

of global cells that country *C* holds for a species *i* where $E_i = \sum_C E_{Ci}$is the total number of expected grid cells for a species across all countries (Fig. 1, top panel). National species stewardship can then be used to weight both the national-level coverage, I_C, and number of species that country *C* is responsible to document, $D_{steward's,C}$ (Fig. 1c):

$$D_{steward's,C} = \sum_{i=1}^{S_C} N_{Ci} ,$$

$$I_C = \frac{1}{D_{steward's,C}} \sum_{i=1}^{S_C} \frac{O_{Ci}}{E_{Ci}} N_{Ci}.$$

In the current form, the SSII is not capable of capturing true absence information. The expected diversity based on species range maps represents summaries over many years and thus a species is not necessarily expected to occur in every cell in every year due to metapopulation and range dynamics, and differences in suitable environmental conditions. This behavior will certainly depress index values, however, we expect this underestimation to be negligible when averaged across sufficiently large numbers of species constituting a taxon. However, we acknowledge that expectations based on expert range maps may vary among species and regions in their accuracy based on available information.

We calculated the SSII for all extant terrestrial vertebrates both with and without consideration of national boundaries. National boundaries were based on the Database of Global Administrative Areas (GADM version 3.6; gadm.org). National boundaries, species range maps, and spatiotemporal biodiversity records were intersected with a global equal-area grid (111 km) using geohash level 5 representations of each dataset. Geohashes are a public domain geocoding system which encodes geographic coordinates with a unique alphanumeric string in a hierarchical structure and known precision. Geohash level 5 represents geographic space with 5 km bounding boxes at the equator that increase in precision as they approach the poles. Geohash level 5 bounding boxes and the respective proportion contained within each geometry were generated for national boundaries, species ranges, and the equal-area grid using the python-geohash library. Spatiotemporal occurrence records were encoded with geohash level 5 using the R package "geohashTools" (24). All three datasets were then intersected based on common geohashes in R 4.0.0 (R Core Team, 2020). The proportion of each grid cell contained within national boundaries was determined based on summing geohash areas weighted by their proportion within each geometry. Grid cells were weighted within data coverage indices based on their proportion within the nation of interest. Biodiversity records were intersected with national grid cells based on shared geohashes which fell fully inside national boundaries. This restriction may eliminate valid records, but avoids erroneously attributing records to nations due to imprecise spatial intersection.

We demonstrated the sensitivity of the SSII to a range of spatial resolutions (110, 55, and 27.5 km) for two example species based on published species distribution models (4) and found that SSII values decrease predictably at finer spatial resolutions (S1 Fig. C, S1 Text).

Species Sampling Effectiveness Index (SSEI)

Species data coverage is determined not just by count of records but also by their complementarity. To estimate the effectiveness of nations' biodiversity data collection, we

computed the evenness of the spatial distribution of records for each species based on Shannon entropy using the R package DescTools (v0.99.36)(25). The Shannon entropy H(X) of a random variable X is an information theoretic metric that measures the expected amount of information or uncertainty in that variable's distribution (26):

$H(X) = -\sum_{x \in \mathcal{X}} P(x) log P(x).$

A uniform distribution ($P(x) = 1/|\mathcal{X}| \; \forall x \in \mathcal{X}$) would have maximum entropy, or evenness, which we denote by $H^*(X) = log(|\mathcal{X}|)$, where $|\mathcal{X}|$ denotes the size of $\mathcal{X}$, the domain of X.

In this application, the entropy $H$of a set of records distributed over Jcells is given by

$$H = -\sum_{j=1}^{J} \frac{n_j}{N} log \frac{n_j}{N},$$

where $n_j$is the number of records in cell j and $N = \Sigma_j n_j$is the total number of records. A uniform distribution would represent spatially even sampling of a species, i.e. the same number of records per grid cell and thus $H^* = log(J)$.

We defined the Species Sampling Effectiveness Index (SSEI) for a species as the ratio H/H^* between the realized evenness of records (i.e. the observed entropy of the distribution of records across all grid cells) and the ideal evenness (i.e. the entropy of a uniform distribution) (Fig. 1c, S1 Fig. D). Similar to SSII, SSEI can be computed at the global, national, or species level and optionally weighted by stewardship at the national level. Global SSEI tracks the ratio of the entropy of the realized and ideal distributions of records for a single species or averaged across many species, without considering national boundaries. National SSEI restricts this calculation to the range cells inside a particular country and takes the average across expected species with data. Steward's SSEI adjusts the National SSEI based on national stewardship of species, as described for the Steward's SSII.

The SSII quantifies the proportion of a species' range with data. The SSEI quantifies how evenly this data is distributed among the grid cells it covers. As formulated, the SSEI metrics penalizes uneven sampling based on the size of the discrepancy of the number of records contained within sampled grid cells (S1 Fig. D). For example, low values of SSEI correspond to situations where a small proportion of grid cells contain many duplicate records and the remaining grid cells contain very few or a single record. In this case we consider ideal sampling to be entirely uniform distribution of records because uneven sampling suggests geographic biases in data collection. The SSEI therefore quantifies the degree of geographic biases within the portion of the range that is sampled. As SSEI is based on normalized entropy, its value does not depend directly on total sampling effort (i.e. total number of records, N), though we note that the range and resolution of the SSEI does depend on N. Thus while SSEI can be compared between species with different overall sampling effort, some care should be taken in interpreting SSEI when sampling effort varies by orders of magnitude or when sampling effort is exceptionally low. Species without data or with data only within a single grid cell are excluded from national averages.

Global trends

We summarized annual trends in the number of occurrence records, the percentage of globally expected species that these records represented, species-level Global SSII and SSEI by class. Statistics were done using R 4.0.0 (27) and the package rstatix (v0.6.0) (28). We tested for the relationship between the proportion of species recorded and SSEI using Spearman's rank correlation.

National trends

We primarily report results for the Steward's SSII, unless otherwise specified. National data coverage indices were calculated for each terrestrial vertebrate group independently and averaged. Recent Steward's SSII and SSEI were determined by averaging values of annual SSII and SSEI over the previous ten years (2010-2019). Recent trends in Steward's SSII and SSEI were determined by testing for significant trends over the previous decade (2010-2019) using linear regression.

We tested for the relationships between variables using Spearman's rank correlation.

We categorized nations into the following four main typologies of status and trends in Steward's SSII over the previous decade: (1) nations with coverage below the global mean and no or decreasing trend; (2) nations with increasing coverage, but below the global mean; (3) nations with coverage above the global mean, but no or decreasing trend; (4) nations with both coverage above the global mean and an increasing trend.

We estimated nations' recent propensity to survey local and highly endemic species as compared to those with larger, multinational ranges by calculating the percentage difference between nation's mean national and Steward's SSII over the previous decade (2010-2019).

S1 Text: Supplementary Text

Scale sensitivity

We demonstrated the potential of the SSII and SSEI to be calculated across a range of spatial resolutions (110, 55, and 27.5 km) for two example species (S1 Fig. C). Because expert-based species distribution maps are not accurate at finer spatial resolutions, we used thresholded output from published species distribution models to estimate ranges at finer resolutions for two hummingbird species, the Glowing puffleg (*Eriocnemis vestita*) and White-sided hillstar (*Oreotrochilus leucopleurus*) (4). Thresholded species distribution model outputs were rescaled to three equal-area grids (110, 55, and 27.5 km) and intersected with records collected over the previous two decades (2000-2019) (S1 Fig. Ca,b). We computed annual SSII and SSEI values over the same time period for both species. Unsurprisingly, data coverage decreased and sampling effectiveness increased at finer spatial resolutions (S1 Fig. Cc). We compared SSII and SSEI values based on each spatial resolution and estimated the slope of relationship for both species independently using linear regression (S1 Fig. Cd-i). For each comparison, we found that 95% confidence intervals of regression slopes overlapped between species, suggesting that relative SSII and SSEI values scale consistently between resolutions among

species. Therefore, while spatial scale clearly impacts the absolute value of the SSII and SSEI, comparisons across species and regions should largely be consistent across spatial resolutions.

Global trends

At the global scale, spatiotemporal species records have grown rapidly over the previous 70 years (1950-2019) (Fig. 3a). Bird species consistently had the largest number of records, with approximately 1000-fold greater number of records collected annually and 3-fold greater percentage of expected species recorded compared to other terrestrial vertebrates (Fig. 3b). The temporal patterns in mean SSII are different, with birds only exceeding the three other groups after 1980, but since then showing near linear-growth in taxon-wide mean SSII and exceeding other classes in 2019 by nearly 10-fold (Fig. 3c).

We compared SSII values among taxa when a comparable number of records were collected (i.e. for a given number of records, how did SSII differ among taxa?). Restricting class-wide SSII values for each class to the years with comparable number of records (42k-102k records; i.e., years 1950s for birds, 1990s to mid 2000s for mammals, and late 2010s for the other two classes), mean SSII was highest for amphibians (0.026), reptiles (0.015), mammals (0.009), and lowest for birds (0.006) (Fig. 3d).

SSEI declined by 6% over the previous two decades for bird species and was highest for reptile species for much of the past 70 years (Fig. 3e). Birds had a negative relationship between the percentage of species recorded and sampling effectiveness (Fig. 3f; Spearman's rho = -0.93, $p < 0.001$).

National trends

Over the previous decade, Steward's SSII varied greatly among nations (Fig. 3a, S1 Table C), with generally higher coverage in Europe, Australia, and the Americas. With the exception of Réunion and Taiwan, all ten nations with the highest data coverage were in Europe.

Steward's SSII has recently increased in a majority of nations (84%), particularly in North America and southern and eastern Europe with nearly half of nations (42%) showing significant ($p < 0.01$) increasing trends (Fig. 3b). Of the minority (13%) with decreasing rates, Finland had the most rapid decrease (-0.021 SSII/year). Despite mostly positive trends, much of Africa and Asia saw only negligible increases in indicator values over the last decade, with the exceptions of India, Sri Lanka, and South Korea which showed large increases in data coverage. Nations were nearly evenly split between either non-significant and significantly increasing Steward's SSII for resident bird species (52.8% and 47.2%, respectively, none decreasing; Fig. 3c). Most nations did not have significant trends in data coverage for mammals (85.8%), amphibians (89.9%), and reptiles (81%).

Recent National SSEI differed strongly among nations (Fig. 4d, S1 Table C). National SSEI was generally lower within western Europe, North America, and Australia. National SSEI and Steward's SSII were weakly, negatively correlated (Spearman's rho = -0.52, $p < 0.001$). A majority of nations (51%) had decreasing SSEI across terrestrial vertebrates, however only 11%

of nations globally had significant ($p < 0.01$), decreasing trends (Fig. 3e). These nations included the United States, Canada, Italy, and South Africa. Decreasing trends in SSEI were most common for bird species (27.5%) (Fig. 4f).

Nearly half (48.6%) of nations had increasing Steward's SSII and decreasing National SSEI (S1 Fig. Aa). Nations' mean SSII over the previous decade generally increased with the total number of biodiversity records collected (S1 Fig. Ab; Spearman's rho = 0.55, $p < 0.001$) and the proportion of expected species recorded (S1 Fig. Ac; Spearman's rho = 0.73, $p < 0.001$). Mean National SSEI generally decreased with the proportion of expected species recorded (S1 Fig. Ad; Spearman's rho = -0.42, $p < 0.001$).

We categorized nations into the following four main types based on Steward's SSII status and trends over the previous decade: (1) coverage below the global mean and no or decreasing trend (42% of nations); (2) increasing coverage, but below the global mean (24%); (3) coverage above the global mean, but no or decreasing trend (17%); (4) both coverage above the global mean and an increasing trend (17%) (Fig. 4a). We highlight national trajectory examples from each group (Fig. 4b). Status and trends in Steward's SSII differed strongly among continents (Fig. 4c).

By comparing National and Steward's SSII, we found that half of nations (50%), incorporating stewardship increased coverage by over 10%, whereas 30.2% showed little change (-10 to 10%) (S1 Fig. B). For some countries (18.8%), incorporating stewardship decreased coverage. For example Steward's SSII was less than half of National SSII for Niger (43.6%) and the Central African Republic (40%), indicating that their endemic or near-endemic species had much lower coverage than other species. In contrast, Steward's SSII strongly exceeded National SSII in small island nations such as Mayotte (227.6%) and Comoros (110.5%), suggesting their high-stewardship species receive particular recording attention.

S1 Text: Supplementary Acknowledgements

Source and credit information for artwork used in figures.

**Figure 1**		
hummingbird silhouette	Margot Michaud [phylopic.org, Public Domain Dedication 1.0]	http://www.phylopic.org/image/b4f64736-abc3-429d-b19e-4c7f74b291c4/
bird silhouette	Chloe Schmidt [phylopic.org, CC BY 3.0]	http://www.phylopic.org/image/be358482-58c7-4ea8-a5ce-e3ef9f4d0db4/
**Figure 2**		
Jaguar	Ashley Lee [Wikimedia, CC BY 4.0]	https://commons.wikimedia.org/wiki/File:Jaguar_Staring_in_the_Distance.jpg
Collared peccary	Charlie Jackson [Wikimedia, CC BY 2.0]	https://commons.wikimedia.org/wiki/File:Collared_Peccary_(49660522358).jpg
**Figures 3 and 4**		
bird silhouette	Chloe Schmidt [phylopic.org, CC BY 3.0]	http://www.phylopic.org/image/be358482-58c7-4ea8-a5ce-e3ef9f4d0db4/
mammal silhouette	David Orr [phylopic.org, Public Domain Mark 1.0]	http://www.phylopic.org/image/da5faa63-085f-4523-a542-e71cb386c999/
amphibian silhouette	Steven Traver [phylopic.org, Public Domain Dedication 1.0]	http://www.phylopic.org/image/4679516b-405b-444f-974d-9775876716e2/
reptile silhouette	Brad McFeeters [phylopic.org, Public Domain Dedication 1.0]	http://www.phylopic.org/image/7dee5849-e764-4694-abf7-d0ae4cc8cabe/

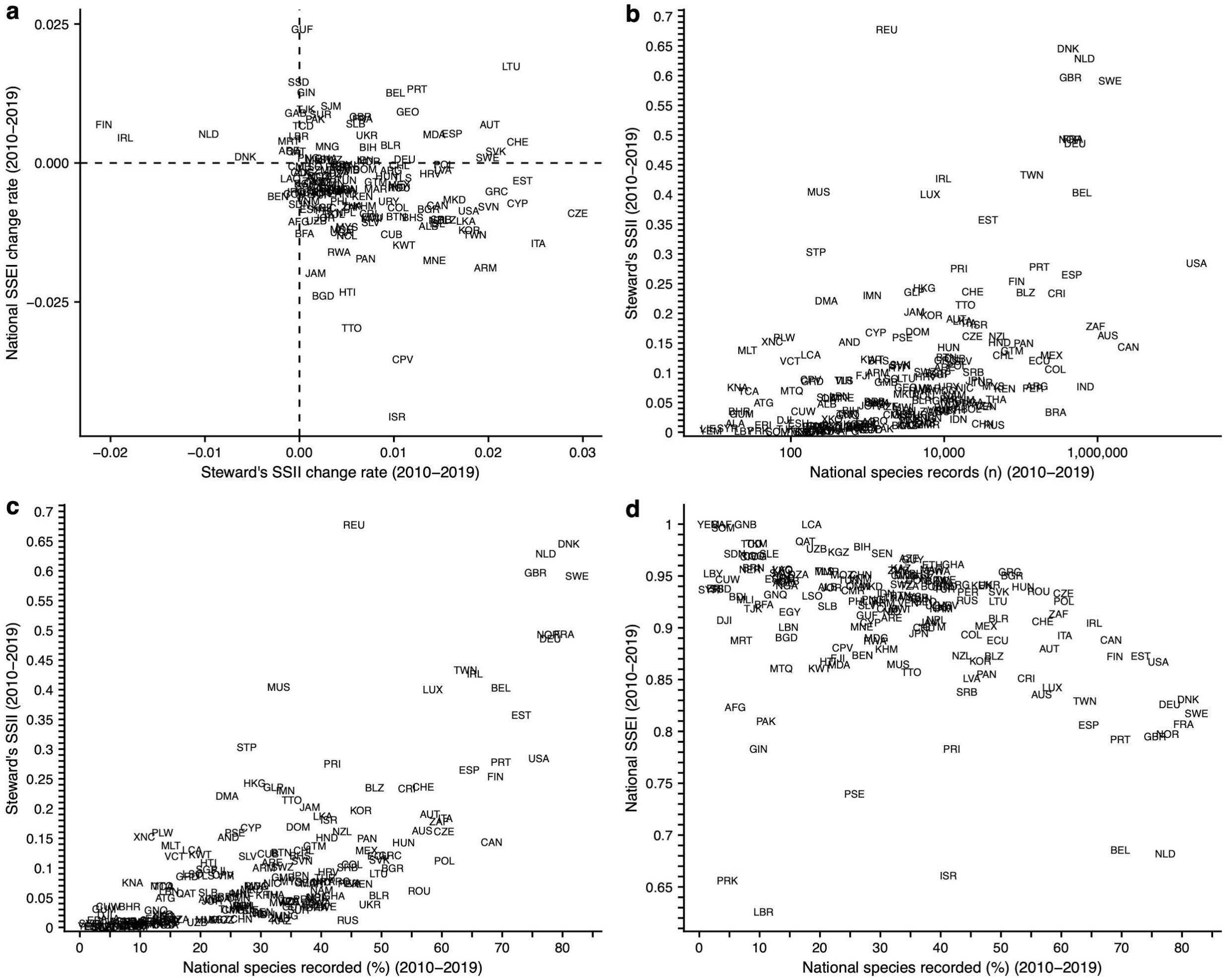

S1 Fig. A. National patterns in data collection, coverage, and sampling effectiveness (2010-2019). a Change rates in Steward's SSII and National SSEI. Dashed lines represent zero slopes. **b-c** Relationship and mismatch between Steward's SSII and total spatiotemporal records collected nationally (**b**) and the percentage of expected species nationally recorded (**c**). **d** Relationship between the percentage of of expected species nationally recorded and mean National SSEI. *The data underlying this Figure may be found in* https://github.com/MapofLife/biodiversity-data-gaps.

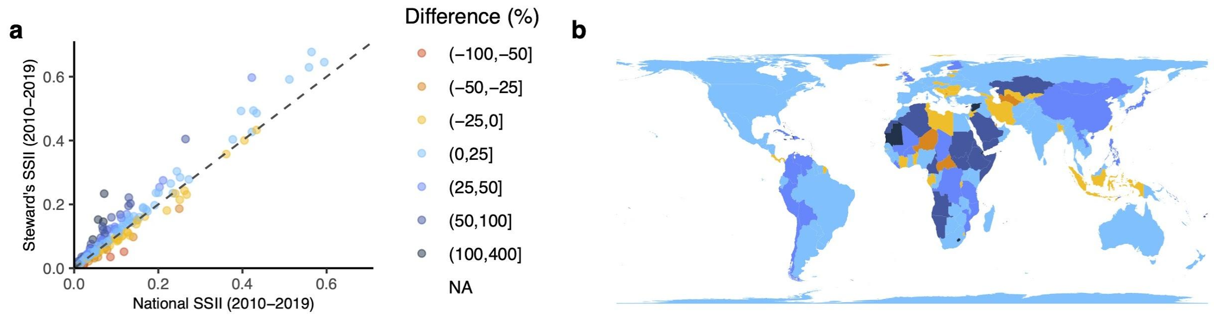

S1 Fig. B. National stewardship in data coverage. a, National and Steward's SSII over the previous decade (2010-2019). Points are colored by the percent difference between national and Steward's SSII. Dashed line represents the 1:1 line between variables. **b,** Relative stewardship of nations, as estimated by percent difference, over the previous decade. Color scale matches that in panel (**a**). *National boundaries from gadm.org. The data underlying this Figure may be found in* https://github.com/MapofLife/biodiversity-data-gaps.

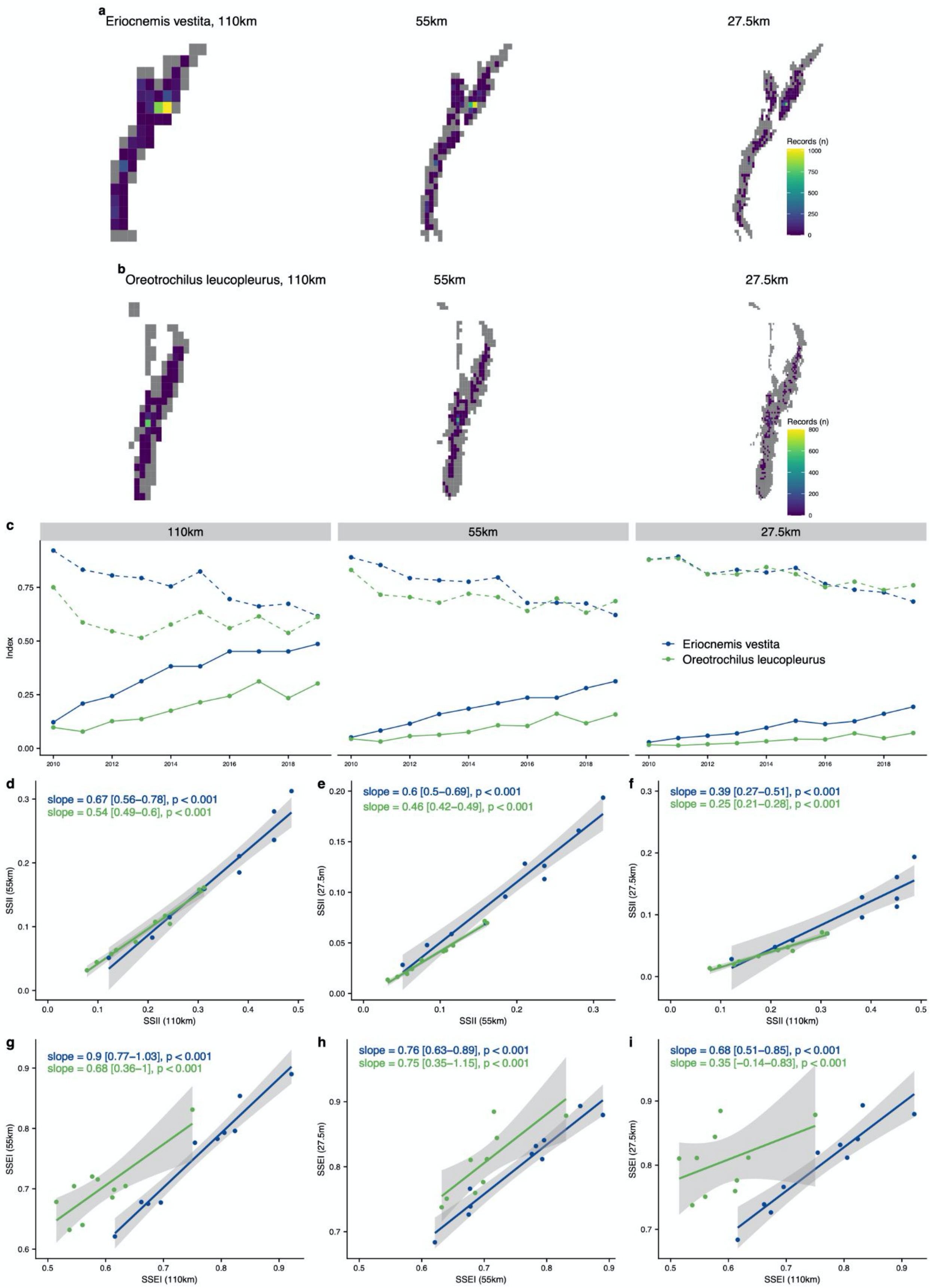

a Eriocnemis vestita, 110km
55km
27.5km
Records (n)
b Oreotrochilus leucopleurus, 110km
55km
27.5km
Records (n)
c
110km
55km
27.5km
Index
Eriocnemis vestita
Oreotrochilus leucopleurus
d
slope = 0.67 [0.56–0.78], p < 0.001
slope = 0.54 [0.49–0.6], p < 0.001
SSII (55km)
SSII (110km)
e
slope = 0.6 [0.5–0.69], p < 0.001
slope = 0.46 [0.42–0.49], p < 0.001
SSII (27.5m)
SSII (55km)
f
slope = 0.39 [0.27–0.51], p < 0.001
slope = 0.25 [0.21–0.28], p < 0.001
SSII (27.5km)
SSII (110km)
g
slope = 0.9 [0.77–1.03], p < 0.001
slope = 0.68 [0.36–1], p < 0.001
SSEI (55km)
SSEI (110km)
h
slope = 0.76 [0.63–0.89], p < 0.001
slope = 0.75 [0.35–1.15], p < 0.001
SSEI (27.5m)
SSEI (55km)
i
slope = 0.68 [0.51–0.85], p < 0.001
slope = 0.35 [−0.14–0.83], p < 0.001
SSEI (27.5km)
SSEI (110km)

S1 Fig. C. Empirical demonstration of the effects of spatial resolution on the SSII and SSEI. a-b, Thresholded species distribution model output (Ellis-Soto et al. 2021) rescaled to three spatial resolutions (110, 55, and 27.5 km) for two hummingbird species, (**a**) the Glowing puffleg (*Eriocnemis vestita*) and (**b**) White-sided hillstar (*Oreotrochilus leucopleurus*). Grid cells are colored by the number of records collected between 2000-2019. **c**, Annual SSII (solid lines) and SSEI (dashed lines) computed at three spatial resolutions. **d-i**, Comparison of SSII (d-f) and SSEI (g-i) values among spatial resolutions (**d,g**: 100 vs. 55 km; **e,h**: 55 vs. 27.5 km; **f,i**: 110 vs. 27.5 km). Grey shading shows 95% confidence interval. Colored text displays slope estimates and 95% confidence intervals for each species (blue: *Eriocnemis vestita*; green: *Oreotrochilus leucopleurus*). *The data underlying this Figure may be found in* https://github.com/MapofLife/biodiversity-data-gaps.

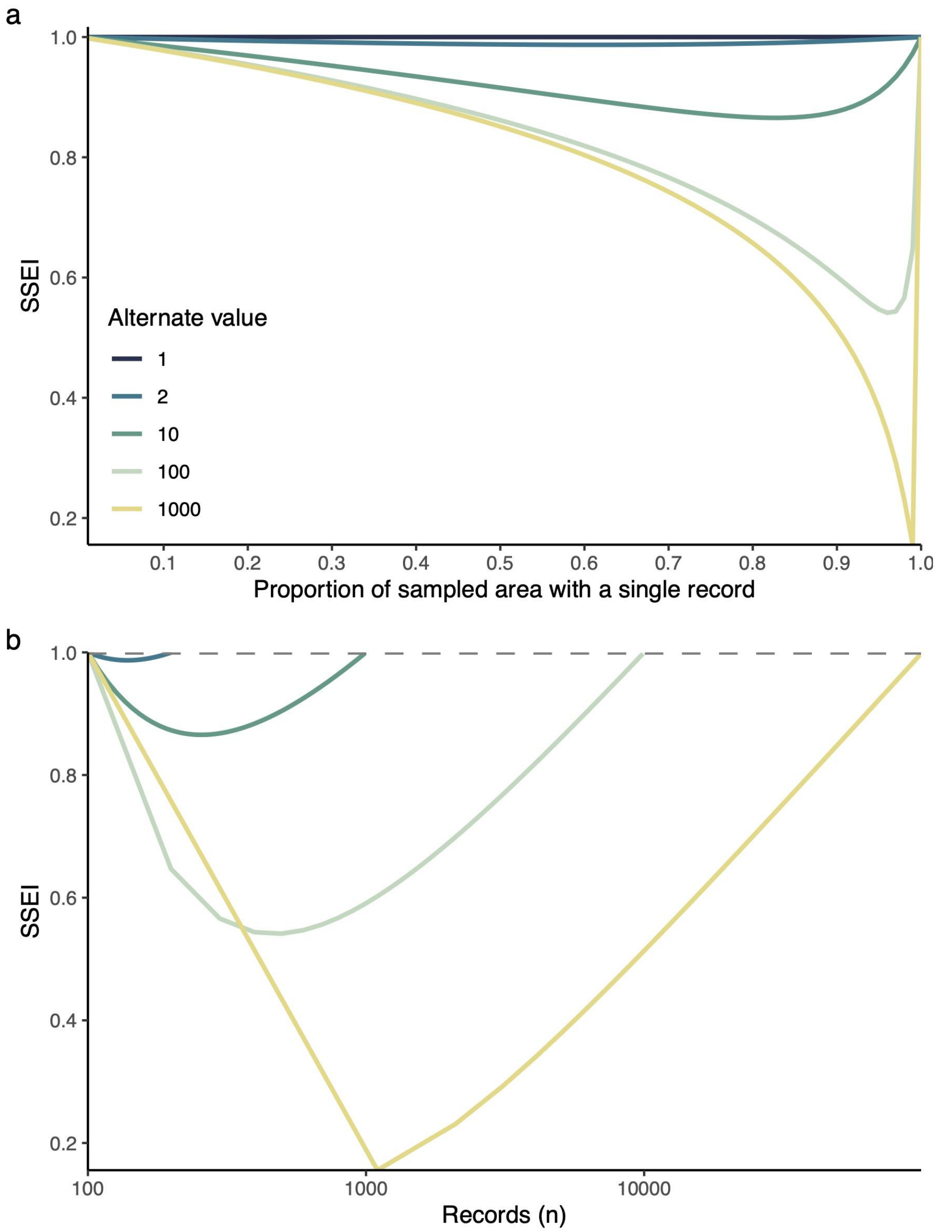

S1 Figure D. Theoretical examples of the Species Sampling Effectiveness Index (SSEI). Each line corresponds to theoretical cases with different levels of evenness of the distribution of biodiversity records for an idealized species with the same range size. In these examples the proportion of the sampled range with a single record vs. alternate values (1, 2, 10, 100, 1000) is adjusted from 0 to 1. SSEI is highest in cases with uniform or near-uniform sampling (i.e., all grid cells either contain one or two records). SSEI is lowest in cases with highly uneven sampling (i.e., a mixture of grid cells with either a single record or 100-1000 records). These examples also highlight that SSEI is identical in the cases where redundant sampling is uniform (i.e., values are the same if all cells have a 1, 10, or 1000 records). Additionally, SSEI approaches the maximum value when only a small minority of cells contain more than a single record (i.e., the proportion of cells with a single record > 90%).

S1 Table A. Species example coverage and sampling effectiveness values. Values presented for the jaguar (*Panthera onca*) and collared peccary (*Pecari tajacu)* as demonstrated in Fig. 2c-e. *The data underlying this Table may be found in* https://github.com/MapofLife/biodiversity-data-gaps.

Species	Year	Records (n)	SSII	SSEI
Panthera onca	2000	8	0.004	0.790
Panthera onca	2001	5	0.004	1.000
Panthera onca	2002	1	0.001	NA
Panthera onca	2003	3	0.001	0.918
Panthera onca	2004	1	0.000	NA
Panthera onca	2005	3	0.002	NA
Panthera onca	2006	5	0.001	0.946
Panthera onca	2007	13	0.004	0.885
Panthera onca	2008	55	0.008	0.652
Panthera onca	2009	17	0.007	0.960
Panthera onca	2010	26	0.011	0.905
Panthera onca	2011	75	0.011	0.851
Panthera onca	2012	23	0.010	0.947
Panthera onca	2013	40	0.011	0.797
Panthera onca	2014	55	0.013	0.868
Panthera onca	2015	62	0.016	0.872
Panthera onca	2016	109	0.016	0.675
Panthera onca	2017	142	0.016	0.691
Panthera onca	2018	45	0.020	0.919
Panthera onca	2019	119	0.020	0.673
Pecari tajacu	2000	121	0.007	0.805
Pecari tajacu	2001	68	0.010	0.795
Pecari tajacu	2002	39	0.006	0.772
Pecari tajacu	2003	20	0.005	0.910
Pecari tajacu	2004	59	0.009	0.744
Pecari tajacu	2005	60	0.008	0.565
Pecari tajacu	2006	156	0.008	0.343
Pecari tajacu	2007	108	0.008	0.698
Pecari tajacu	2008	107	0.013	0.817
Pecari tajacu	2009	96	0.011	0.682
Pecari tajacu	2010	123	0.013	0.708
Pecari tajacu	2011	299	0.009	0.647
Pecari tajacu	2012	287	0.014	0.712
Pecari tajacu	2013	188	0.021	0.618
Pecari tajacu	2014	137	0.020	0.847
Pecari tajacu	2015	209	0.031	0.701
Pecari tajacu	2016	155	0.026	0.852
Pecari tajacu	2017	212	0.040	0.883
Pecari tajacu	2018	278	0.042	0.888
Pecari tajacu	2019	343	0.044	0.860

S1 Table B. National example data coverage and sampling effectiveness values. Values presented for the jaguar (*Panthera onca*) and collared peccary (*Pecari tajacu)* as demonstrated in Fig. 2f-g. *The data underlying this Table may be found in* https://github.com/MapofLife/biodiversity-data-gaps.

Country	Year	National SSII	Steward's SSII	National SSEI	Steward's SSEI
Brazil	2000	0.001	0.001	0.875	0.011
Brazil	2001	0.000	0.000	1.000	0.003
Brazil	2002	0.001	0.001	0.982	0.004
Brazil	2003	0.001	0.001	0.953	0.005
Brazil	2004	0.000	0.000	0.968	0.005
Brazil	2005	0.000	0.000	0.961	0.009
Brazil	2006	0.000	0.000	1.000	0.005
Brazil	2007	0.002	0.002	0.911	0.011
Brazil	2008	0.001	0.001	0.969	0.009
Brazil	2009	0.001	0.001	0.933	0.017
Brazil	2010	0.003	0.003	0.977	0.009
Brazil	2011	0.001	0.001	0.972	0.028
Brazil	2012	0.002	0.002	0.978	0.027
Brazil	2013	0.006	0.006	0.958	0.033
Brazil	2014	0.002	0.002	0.965	0.034
Brazil	2015	0.004	0.004	0.930	0.041
Brazil	2016	0.006	0.006	0.944	0.040
Brazil	2017	0.006	0.006	0.896	0.048
Brazil	2018	0.011	0.011	0.936	0.051
Brazil	2019	0.012	0.012	0.897	0.077
Colombia	2000	0.006	0.005	0.915	0.015
Colombia	2001	0.006	0.005	0.932	0.031
Colombia	2002	0.000	0.000	0.929	0.028
Colombia	2003	0.000	0.000	0.920	0.034
Colombia	2004	0.000	0.000	0.938	0.038
Colombia	2005	0.000	0.000	0.968	0.040
Colombia	2006	0.006	0.005	0.916	0.019
Colombia	2007	0.015	0.014	0.866	0.042
Colombia	2008	0.022	0.022	0.933	0.043
Colombia	2009	0.023	0.022	0.908	0.057
Colombia	2010	0.018	0.017	0.924	0.050
Colombia	2011	0.046	0.050	0.883	0.048
Colombia	2012	0.039	0.037	0.891	0.084
Colombia	2013	0.040	0.037	0.901	0.087
Colombia	2014	0.012	0.012	0.869	0.129
Colombia	2015	0.071	0.067	0.849	0.135
Colombia	2016	0.039	0.039	0.878	0.111
Colombia	2017	0.054	0.050	0.793	0.094
Colombia	2018	0.085	0.082	0.849	0.110
Colombia	2019	0.028	0.026	0.859	0.075

Country	Year	National SSII	Steward's SSII	National SSEI	Steward's SSEI
Costa Rica	2000	0.009	0.008	1.000	0.004
Costa Rica	2001	0.000	0.000	0.923	0.014
Costa Rica	2002	0.000	0.000	NA	0.000
Costa Rica	2003	0.000	0.000	1.000	0.004
Costa Rica	2004	0.000	0.000	NA	0.000
Costa Rica	2005	0.000	0.000	1.000	0.012
Costa Rica	2006	0.000	0.000	0.918	0.004
Costa Rica	2007	0.000	0.000	0.940	0.018
Costa Rica	2008	0.129	0.107	0.907	0.021
Costa Rica	2009	0.223	0.217	0.956	0.022
Costa Rica	2010	0.000	0.000	0.927	0.029
Costa Rica	2011	0.094	0.110	0.900	0.039
Costa Rica	2012	0.142	0.117	0.813	0.029
Costa Rica	2013	0.248	0.246	0.838	0.033
Costa Rica	2014	0.223	0.185	0.905	0.064
Costa Rica	2015	0.238	0.234	0.906	0.050
Costa Rica	2016	0.000	0.000	0.779	0.037
Costa Rica	2017	0.212	0.176	0.830	0.059
Costa Rica	2018	0.215	0.178	0.856	0.064
Costa Rica	2019	0.373	0.350	0.868	0.092
Mexico	2000	0.031	0.030	0.869	0.087
Mexico	2001	0.005	0.006	0.871	0.079
Mexico	2002	0.013	0.012	0.797	0.072
Mexico	2003	0.009	0.010	0.892	0.074
Mexico	2004	0.015	0.017	0.888	0.127
Mexico	2005	0.026	0.030	0.884	0.096
Mexico	2006	0.014	0.015	0.928	0.077
Mexico	2007	0.022	0.023	0.845	0.059
Mexico	2008	0.062	0.061	0.877	0.109
Mexico	2009	0.048	0.050	0.847	0.100
Mexico	2010	0.081	0.080	0.895	0.103
Mexico	2011	0.026	0.024	0.786	0.056
Mexico	2012	0.060	0.057	0.941	0.076
Mexico	2013	0.029	0.028	0.935	0.090
Mexico	2014	0.139	0.132	0.930	0.091
Mexico	2015	0.153	0.150	0.872	0.118
Mexico	2016	0.129	0.132	0.872	0.144
Mexico	2017	0.161	0.168	0.906	0.177
Mexico	2018	0.145	0.151	0.903	0.161
Mexico	2019	0.141	0.150	0.857	0.172

S1 Table C. National data coverage and sampling effectiveness values over the previous decade (2010-2019). ISO3 codes and mean values for National and Steward's SSII and SSEI for nations. *The data underlying this Table may be found in* https://github.com/MapofLife/biodiversity-data-gaps.

Country	National SSII	Steward's SSII	National SSEI	Steward's SSEI	ISO3
Afghanistan	0.002	0.002	0.823	0.820	AFG
Åland	0.012	0.015	NA	NA	ALA
Albania	0.056	0.048	0.940	0.931	ALB
Algeria	0.008	0.013	0.952	0.969	DZA
Andorra	0.149	0.152	NA	NA	AND
Angola	0.005	0.008	0.944	0.949	AGO
Antarctica	0.001	0.001	0.876	0.875	ATA
Antigua and Barbuda	0.059	0.050	NA	NA	ATG
Argentina	0.076	0.077	0.942	0.946	ARG
Armenia	0.093	0.100	0.924	0.933	ARM
Australia	0.142	0.163	0.836	0.837	AUS
Austria	0.164	0.191	0.880	0.869	AUT
Azerbaijan	0.049	0.043	0.967	0.973	AZE
Bahamas	0.078	0.120	0.953	0.958	BHS
Bahrain	0.056	0.035	NA	NA	BHR
Bangladesh	0.015	0.011	0.891	0.886	BGD
Barbados	0.118	0.052	NA	NA	BRB
Belarus	0.050	0.054	0.909	0.909	BLR
Belgium	0.377	0.404	0.686	0.708	BEL
Belize	0.235	0.235	0.873	0.864	BLZ
Benin	0.043	0.037	0.874	0.884	BEN
Bhutan	0.102	0.126	0.931	0.939	BTN
Bolivia	0.029	0.040	0.940	0.934	BOL
Bosnia and Herzegovina	0.047	0.037	0.978	0.982	BIH
Botswana	0.036	0.042	0.955	0.957	BWA
Bouvet Island	0.000	0.000	NA	NA	BVT
Brazil	0.029	0.034	0.945	0.945	BRA
Brunei	0.022	0.009	0.958	0.959	BRN
Bulgaria	0.106	0.100	0.951	0.958	BGR
Burkina Faso	0.004	0.005	0.923	0.919	BFA
Burundi	0.012	0.010	0.930	0.924	BDI
Cambodia	0.054	0.054	0.879	0.889	KHM
Cameroon	0.019	0.029	0.936	0.934	CMR
Canada	0.127	0.143	0.888	0.893	CAN
Cape Verde	0.084	0.089	0.881	0.870	CPV
Central African Republic	0.001	0.001	1.000	1.000	CAF
Chad	0.002	0.002	0.981	0.983	TCD
Chile	0.097	0.130	0.900	0.905	CHL
China	0.010	0.014	0.951	0.949	CHN
Colombia	0.075	0.106	0.894	0.878	COL

Country	National SSII	Steward's SSII	National SSEI	Steward's SSEI	ISO3
Comoros	0.069	0.146	0.985	0.985	COM
Costa Rica	0.240	0.234	0.851	0.840	CRI
Côte d'Ivoire	0.003	0.002	0.969	0.975	CIV
Croatia	0.097	0.093	0.922	0.894	HRV
Cuba	0.086	0.124	0.919	0.918	CUB
Curaçao	0.018	0.035	0.947	0.935	CUW
Cyprus	0.110	0.168	0.905	0.897	CYP
Czech Republic	0.168	0.162	0.933	0.938	CZE
Democratic Republic of the Congo	0.003	0.004	0.948	0.953	COD
Denmark	0.594	0.645	0.831	0.837	DNK
Djibouti	0.009	0.021	0.908	0.854	DJI
Dominica	0.130	0.221	NA	NA	DMA
Dominican Republic	0.125	0.169	0.946	0.944	DOM
Ecuador	0.111	0.120	0.888	0.885	ECU
Egypt	0.013	0.014	0.915	0.923	EGY
El Salvador	0.100	0.120	0.922	0.925	SLV
Equatorial Guinea	0.027	0.029	0.932	0.930	GNQ
Eritrea	0.007	0.012	0.938	0.941	ERI
Estonia	0.361	0.358	0.873	0.874	EST
Ethiopia	0.024	0.038	0.961	0.945	ETH
Falkland Islands	0.027	0.032	1.000	1.000	FLK
Faroe Islands	0.086	0.035	0.955	0.958	FRO
Fiji	0.063	0.095	0.871	0.847	FJI
Finland	0.203	0.254	0.872	0.875	FIN
France	0.426	0.494	0.807	0.811	FRA
French Guiana	0.034	0.030	0.912	0.922	GUF
French Polynesia	0.114	0.101	0.933	0.886	PYF
French Southern Territories	0.013	0.013	0.713	0.713	ATF
Gabon	0.011	0.010	0.945	0.940	GAB
Gambia	0.102	0.085	0.951	0.941	GMB
Georgia	0.077	0.075	0.925	0.918	GEO
Germany	0.434	0.486	0.826	0.826	DEU
Ghana	0.052	0.053	0.961	0.965	GHA
Greece	0.105	0.121	0.954	0.951	GRC
Greenland	0.002	0.002	0.960	0.957	GRL
Grenada	0.083	0.086	NA	NA	GRD
Guadeloupe	0.194	0.236	NA	NA	GLP
Guam	0.048	0.031	NA	NA	GUM
Guatemala	0.124	0.137	0.901	0.893	GTM
Guinea	0.006	0.006	0.783	0.776	GIN

Country	National SSII	Steward's SSII	National SSEI	Steward's SSEI	ISO3
Guinea–Bissau	0.010	0.011	1.000	1.000	GNB
Guyana	0.033	0.035	0.966	0.966	GUY
Haiti	0.072	0.109	0.868	0.868	HTI
Heard Island and McDonald Islands	0.042	0.048	NA	NA	HMD
Honduras	0.130	0.151	0.940	0.923	HND
Hong Kong	0.262	0.243	NA	NA	HKG
Hungary	0.138	0.143	0.940	0.943	HUN
Iceland	0.250	0.186	0.893	0.892	ISL
India	0.064	0.077	0.946	0.943	IND
Indonesia	0.025	0.022	0.933	0.934	IDN
Iran	0.026	0.024	0.926	0.926	IRN
Iraq	0.018	0.019	0.948	0.950	IRQ
Ireland	0.422	0.427	0.905	0.908	IRL
Isle of Man	0.267	0.231	NA	NA	IMN
Israel	0.220	0.181	0.661	0.666	ISR
Italy	0.171	0.184	0.893	0.891	ITA
Jamaica	0.134	0.203	0.906	0.901	JAM
Japan	0.066	0.088	0.895	0.894	JPN
Jordan	0.048	0.045	0.939	0.940	JOR
Kazakhstan	0.007	0.011	0.957	0.956	KAZ
Kenya	0.065	0.073	0.941	0.930	KEN
Kiribati	0.000	0.000	NA	NA	KIR
Kosovo	0.034	0.022	0.955	0.957	XKO
Kuwait	0.097	0.123	0.861	0.862	KWT
Kyrgyzstan	0.014	0.014	0.973	0.979	KGZ
Laos	0.013	0.018	0.956	0.957	LAO
Latvia	0.072	0.075	0.851	0.852	LVA
Lebanon	0.047	0.061	0.901	0.880	LBN
Lesotho	0.044	0.090	0.931	0.929	LSO
Liberia	0.008	0.011	0.626	0.657	LBR
Libya	0.002	0.002	0.952	0.957	LBY
Liechtenstein	0.009	0.006	NA	NA	LIE
Lithuania	0.103	0.090	0.926	0.929	LTU
Luxembourg	0.405	0.400	0.843	0.835	LUX
Macedonia	0.074	0.064	0.940	0.952	MKD
Madagascar	0.063	0.070	0.890	0.887	MDG
Malawi	0.038	0.042	0.918	0.922	MWI
Malaysia	0.076	0.077	0.930	0.938	MYS
Mali	0.002	0.002	0.928	0.940	MLI
Malta	0.148	0.138	NA	NA	MLT

Country	National SSII	Steward's SSII	National SSEI	Steward's SSEI	ISO3
Martinique	0.084	0.070	0.861	0.809	MTQ
Mauritania	0.005	0.010	0.888	0.864	MRT
Mauritius	0.265	0.405	0.865	0.865	MUS
Mayotte	0.071	0.234	NA	NA	MYT
Mexico	0.114	0.129	0.902	0.896	MEX
Micronesia	0.063	0.083	NA	NA	FSM
Moldova	0.055	0.049	0.865	0.828	MDA
Mongolia	0.014	0.020	0.950	0.949	MNG
Montenegro	0.070	0.058	0.901	0.915	MNE
Morocco	0.063	0.074	0.956	0.953	MAR
Mozambique	0.010	0.013	0.951	0.953	MOZ
Myanmar	0.011	0.013	0.954	0.952	MMR
Namibia	0.038	0.063	0.919	0.923	NAM
Nepal	0.050	0.051	0.908	0.929	NPL
Netherlands	0.558	0.629	0.682	0.675	NLD
New Caledonia	0.117	0.143	0.925	0.922	NCL
New Zealand	0.131	0.162	0.873	0.864	NZL
Nicaragua	0.064	0.075	0.916	0.913	NIC
Niger	0.003	0.002	0.956	0.957	NER
Nigeria	0.004	0.005	0.941	0.944	NGA
Niue	0.028	0.032	NA	NA	NIU
North Korea	0.002	0.003	0.656	0.656	PRK
Northern Cyprus	0.057	0.152	NA	NA	XNC
Norway	0.397	0.493	0.798	0.806	NOR
Oman	0.048	0.050	0.940	0.923	OMN
Pakistan	0.005	0.006	0.810	0.813	PAK
Palau	0.089	0.159	NA	NA	PLW
Palestina	0.155	0.160	0.740	0.704	PSE
Panama	0.151	0.150	0.855	0.854	PAN
Papua New Guinea	0.022	0.023	0.927	0.923	PNG
Paraguay	0.044	0.048	0.948	0.950	PRY
Peru	0.059	0.074	0.934	0.925	PER
Philippines	0.037	0.056	0.926	0.927	PHL
Poland	0.101	0.112	0.926	0.924	POL
Portugal	0.273	0.278	0.792	0.789	PRT
Puerto Rico	0.211	0.275	0.783	0.777	PRI
Qatar	0.078	0.058	0.983	0.980	QAT
Republic of Congo	0.003	0.004	0.969	0.971	COG
Reunion	0.564	0.677	NA	NA	REU
Romania	0.065	0.062	0.935	0.931	ROU

Country	National SSII	Steward's SSII	National SSEI	Steward's SSEI	ISO3
Russia	0.012	0.012	0.927	0.922	RUS
Rwanda	0.078	0.070	0.887	0.888	RWA
Saint Helena	0.023	0.011	NA	NA	SHN
Saint Kitts and Nevis	0.040	0.076	NA	NA	KNA
Saint Lucia	0.067	0.130	1.000	1.000	LCA
Saint Pierre and Miquelon	0.015	0.017	NA	NA	SPM
Saint Vincent and the Grenadines	0.069	0.120	NA	NA	VCT
Samoa	0.129	0.195	0.918	0.937	WSM
São Tomé and Príncipe	0.244	0.303	NA	NA	STP
Saudi Arabia	0.006	0.010	0.953	0.956	SAU
Senegal	0.026	0.026	0.972	0.971	SEN
Serbia	0.114	0.101	0.838	0.840	SRB
Sierra Leone	0.011	0.012	0.972	0.973	SLE
Singapore	0.140	0.098	NA	NA	SGP
Slovakia	0.126	0.113	0.935	0.942	SVK
Slovenia	0.128	0.112	0.928	0.928	SVN
Solomon Islands	0.040	0.059	0.921	0.907	SLB
Somalia	0.001	0.001	0.997	0.994	SOM
South Africa	0.174	0.178	0.913	0.913	ZAF
South Georgia and the South Sandwich Islands	0.112	0.157	1.000	1.000	SGS
South Korea	0.191	0.197	0.868	0.884	KOR
South Sudan	0.001	0.002	0.938	0.945	SSD
Spain	0.230	0.265	0.806	0.816	ESP
Sri Lanka	0.164	0.187	0.920	0.910	LKA
Sudan	0.001	0.001	0.971	0.970	SDN
Suriname	0.028	0.030	0.929	0.946	SUR
Svalbard and Jan Mayen	0.097	0.093	0.777	0.712	SJM
Swaziland	0.132	0.102	0.942	0.941	SWZ
Sweden	0.511	0.591	0.817	0.840	SWE
Switzerland	0.200	0.236	0.906	0.898	CHE
Syria	0.003	0.007	0.937	0.952	SYR
Taiwan	0.433	0.433	0.829	0.843	TWN
Tajikistan	0.005	0.005	0.919	0.917	TJK
Tanzania	0.032	0.045	0.941	0.936	TZA
Thailand	0.051	0.055	0.929	0.931	THA
Timor−Leste	0.066	0.088	0.955	0.942	TLS
Togo	0.009	0.009	0.970	0.977	TGO
Tonga	0.003	0.000	NA	NA	TON
Trinidad and Tobago	0.251	0.214	0.857	0.867	TTO
Tunisia	0.031	0.031	0.946	0.943	TUN

Country	National SSII	Steward's SSII	National SSEI	Steward's SSEI	ISO3
Turkey	0.080	0.084	0.938	0.938	TUR
Turkmenistan	0.002	0.001	0.981	0.985	TKM
Turks and Caicos Islands	0.056	0.069	NA	NA	TCA
Uganda	0.048	0.051	0.922	0.927	UGA
Ukraine	0.036	0.039	0.942	0.949	UKR
United Arab Emirates	0.105	0.110	0.910	0.914	ARE
United Kingdom	0.422	0.597	0.795	0.803	GBR
United States	0.252	0.284	0.867	0.875	USA
Uruguay	0.070	0.077	0.940	0.942	URY
Uzbekistan	0.010	0.008	0.976	0.978	UZB
Vanuatu	0.040	0.049	0.973	0.978	VUT
Venezuela	0.031	0.044	0.924	0.905	VEN
Vietnam	0.031	0.037	0.948	0.940	VNM
Virgin Islands, U.S.	0.105	0.087	NA	NA	VIR
Western Sahara	0.008	0.015	0.947	0.945	ESH
Yemen	0.002	0.002	1.000	1.000	YEM
Zambia	0.015	0.016	0.955	0.951	ZMB
Zimbabwe	0.033	0.035	0.947	0.944	ZWE

References

1. Gaston KJ, Fuller RA. The sizes of species' geographic ranges. Journal of Applied Ecology. 2009;46(1):1–9.

2. Hurlbert AH, Jetz W. Species richness, hotspots, and the scale dependence of range maps in ecology and conservation. Proceedings of the National Academy of Sciences. 2007 Aug 14;104(33):13384–9.

3. Jetz W, McPherson JM, Guralnick RP. Integrating biodiversity distribution knowledge: toward a global map of life. Trends in Ecology & Evolution. 2012 Mar 1;27(3):151–9.

4. Ellis-Soto D, Merow C, Amatulli G, Parra JL, Jetz W. Continental-scale 1 km hummingbird diversity derived from fusing point records with lateral and elevational expert information. Ecography. 2021 April;44(4):640-652. Available from: https://onlinelibrary.wiley.com/doi/abs/10.1111/ecog.05119

5. Jetz W, Thomas GH, Joy JB, Hartmann K, Mooers AO. The global diversity of birds in space and time. Nature. 2012 Nov;491(7424):444–8.

6. IUCN. International Union for Conservation of Nature - Red List of Threatened Species. 2017.

7. IUCN. International Union for Conservation of Nature - Red List of Threatened Species. 2016.

8. Roll U, Feldman A, Novosolov M, Allison A, Bauer AM, Bernard R, et al. The global distribution of tetrapods reveals a need for targeted reptile conservation. Nature Ecology & Evolution. 2017 Nov;1(11):1677–82.

9. Chen I-C, Hill JK, Ohlemüller R, Roy DB, Thomas CD. Rapid Range Shifts of Species Associated with High Levels of Climate Warming. Science. 2011 Aug 19;333(6045):1024–6.

10. Lenoir J, Svenning J-C. Climate-related range shifts – a global multidimensional synthesis and new research directions. Ecography. 2015;38(1):15–28.

11. Lenoir J, Bertrand R, Comte L, Bourgeaud L, Hattab T, Murienne J, et al. Species better track climate warming in the oceans than on land. Nat Ecol Evol. 2020 Aug;4(8):1044–59.

12. Parmesan C, Yohe G. A globally coherent fingerprint of climate change impacts across natural systems. Nature. 2003 Jan;421(6918):37–42.

13. Pecl GT, Araújo MB, Bell JD, Blanchard J, Bonebrake TC, Chen I-C, et al. Biodiversity redistribution under climate change: Impacts on ecosystems and human well-being. Science. 2017 Mar 31;355(6332):eaai9214.

14. Guo F, Lenoir J, Bonebrake TC. Land-use change interacts with climate to determine elevational species redistribution. Nat Commun. 2018 Dec;9(1):1315.

15. Sirami C, Caplat P, Popy S, Clamens A, Arlettaz R, Jiguet F, et al. Impacts of global change on species distributions: obstacles and solutions to integrate climate and land use. Global Ecology and Biogeography. 2017;26(4):385–94.

16. GBIF.org. GBIF Occurrence Download https://doi.org/10.15468/dl.5gzpc3. 2020 Apr 11;

17. Clements JF, Schulenberg TS, Iliff MJ, Billerman SM, Fredericks TA, Sullivan BL, et al. The eBird/Clements Checklist of Birds of the World: v2019 [Internet]. 2019. Available from: https://www.birds.cornell.edu/clementschecklist/download/

18. Upham NS, Esselstyn JA, Jetz W. Inferring the mammal tree: Species-level sets of phylogenies for questions in ecology, evolution, and conservation. PLOS Biology. 2019 Dec 4;17(12):e3000494.

19. AmphibiaWeb: Information on amphibian biology and conservation [Internet]. 2016. Available from: http://amphibiaweb.org

20. Uetz P, Freed P, Hosek J. The Reptile Database. 2019; Available from: http://www.reptile-database.org

21. Avibase - The World Bird Database [Internet]. Available from: http://avibase.bsc-eoc.org

22. Integrated Taxonomic Information System on-line database [Internet]. Available from: http://www.itis.gov

23. Wilson DE, Reeder DM. Mammal Species of the World. A Taxonomic and Geographic Reference (3rd ed). Vol. 2. Johns Hopkins University Press; 2005.

24. Chirico M. geohashTools: Tools for Working with Geohashes. 2020; Available from: https://CRAN.R-project.org/package=geohashTools

25. Signorell A. DescTools: Tools for descriptive statistics. R package version 0.99.36. 2020; Available from: https://cran.r-project.org/package=DescTools

26. Shannon CE. A mathematical theory of communication. The Bell System Technical Journal. 1948 Jul;27(3):379–423.

27. R Core Team. R: A Language and Environment for Statistical Computing. 2020;

28. Kassambra A. rstatix: Pipe-Friendly Framework for Basic Statistical Tests. R package version 0.6.0. 2020; Available from: https://CRAN.R-project.org/package=rstatix
