# Supplementary material for: Global and national trends, gaps, and opportunities in documenting and monitoring species distributions
Source: PLoS Biol. 2021 Aug 12;19(8):e3001336. doi: 10.1371/journal.pbio.3001336 (PMC8360587; doi:10.1371/journal.pbio.3001336)
Supplement: Attachment — Submitted filename: Oliver_etal_SSII_reviews.pdf [file pbio.3001336.s002.pdf]

Dear Dr. Oliver,

Thank you very much for submitting your manuscript "Global and national trends in documenting and monitoring species distributions" for consideration as a Research Article at PLOS Biology. Your manuscript has been evaluated by the PLOS Biology editors, an Academic Editor with relevant expertise, and by four independent reviewers. Many thanks for your patience over the holiday period.

You'll see that the assessments are broadly very positive, but there are multiple requests for you to justify and/or conduct sensitivity analysis on the choice of grid size. There are several further substantial requests, regarding treatment of empty cells, treatment of migrant species, data and code availability (please see PLOS' Data Policy, which is quite stringent), methodological clarifications and statements of limitations. Reviewer #4 also feels that the SSEI metric may be flawed and not truly independent from the SSII, making it redundant over quite a swathe of parameter space; you hould address or rebut this. The Academic Editor asked me to draw your attention to points 1 and 2 from reviewer #1, and the multiple calls for data availability (which we will enforce).

In light of the reviews (below), we will not be able to accept the current version of the manuscript, but we would welcome re-submission of a much-revised version that takes into account the reviewers' comments. We cannot make any decision about publication until we have seen the revised manuscript and your response to the reviewers' comments. Your revised manuscript is also likely to be sent for further evaluation by the reviewers.

Thank you again for your submission to our journal. We hope that our editorial process has been constructive thus far, and we welcome your feedback at any time. Please don't hesitate to contact us if you have any questions or comments.

Sincerely,
Roli Roberts

Dear Dr. Roberts,

We sincerely thank you, the Associate Editor, and reviewers for your time and thoughtful consideration. We feel the reviewers' suggestions have greatly improved the manuscript. Below, please find our detailed responses to each of the reviewers' comments. We have conducted the suggested sensitivity analysis and included it as Supplementary Figure 3. We have also performed additional demonstrations of the SSEI metric (Supplementary Figure 4b) and provided additional clarifying text. In general, we have added text to address the requests related to methodological developments and limitations and updated our data availability statement. As requested, we have added a glossary of relevant terms to Figure 1, if permitted by the format of this article.
We thank you for your further consideration of our work.

Best,
Ruth Oliver

Complete reviews

Reviewer #1:

It was a pleasure to read the manuscript titled "Global and national trends in documenting and monitoring species distributions". I actually saw this posted as a preprint, so getting the opportunity to review it afforded me to take the time and dive in more than I would have as it was in my 'to read' pile.

Overall, I fully support the notion behind what the authors are highlighting here. This is best captured by the idea that 'more data' does not necessarily mean more informed biodiversity decisions and policies (line 61). We are getting more data now than ever before, but are we any better off for it? Who knows! I agree that the general approach to provide quantitative metrics to track a given nation's progress is important. I also think that this could be a method for countries to hold one another accountable in terms of their contributions to biodiversity sampling. I think this paper is suitable for PLoS Biology, and will generate broad interest for an international audience no doubt. However, I think there are a few concerns that if addressed, could significantly strengthen this manuscript. Here I have a few 'major concerns' and then some minor concerns after this. I admit these are somewhat substantial concerns/comments/requests. But in all honesty, I think this has major potential, so would hate to see it published without highlighting the full potential of what you are doing. It will be interesting to see what other Reviewers think about this one, but regardless, I hope these comments are helpful for you!

1) Although I fully support the notion here and behind this paper, I am on the fence about this paper as it currently stands. This is because it currently reads as 50% a methods paper and 50% interpretation of the results. I think the SSII and SSEI are interesting and novel, but the derivation is buried in the methods and not given proper thought at times (e.g., in regard to potential biases that are mentioned in passing). And because of this, I think the manuscript suffers because I am left wanting more of the method development and showing that it truly will work, but also left wanting more of the interpretation and what countries are doing better or worst etc. etc. This is highlighted most in line 240. I really think this would be much stronger if the authors could show that indeed, the metric can be extended at different spatial grains. I would recommend a case study of a few species and going down by the size of the grid, as my current worries is that the 110 km is just far too large for a lot of species. (See next point).

We appreciate the reviewer’s comment regarding the manuscript’s challenges in presenting both methodological development and empirical assessment in a single paper. In general, we feel the combined presentation of new methodology alongside empirical assessment is one of this manuscript’s key strengths. For this field in particular, we feel new methodologies disconnected from applications are of limited use where assessment frameworks and decision-makers critically depend on more sophisticated methods. We have added supporting text and supplementary figures throughout the manuscript to better clarify the methods and potential biases.

In response to the reviewer’s comment regarding the statement on L240 (copied below), we have updated the text to clarify that the SSII in its current format already characterizes data coverage across

scales (species, national, global), but that the framework and indices are capable of extending to other spatiotemporal grains, taxa, and data types.

We have followed the reviewer's suggestion and have provided empirical examples of how the SSII can be extended to finer spatial resolutions for two example species (Supplementary Fig. 3). We have added text throughout the manuscript to clarify this point.

Original text (L240):
With the aforementioned limitations and the further potential to extend the metric to account for different spatiotemporal grains, taxa, and data types in mind, the SSII offers an effective initial characterization of biodiversity information at the species, national, and global scales.

Updated text (L240):
The SSII offers an effective initial characterization of biodiversity information at the species, national, and global scales, with the potential to extend the metric to account for different spatiotemporal grains (Supplementary Fig. 3), taxa, and data types.

Additional text (Main text):
Our methodology and analysis infrastructure is capable of flexibly accommodating different spatial resolutions (Supplementary Fig. 3, Supplementary Text) as more precise information on species' ranges becomes available (e.g. through species distribution modeling) for a broader range of taxa.

Original text (Methods, Species distribution data):
Spatial biodiversity data coverage, hereafter referred to as data coverage, can be calculated at any spatial, temporal, or taxonomic resolution of interest. Doing so requires an estimation of expected biodiversity. We estimated terrestrial vertebrate diversity based on composites of single species distribution maps, which have been shown to approximate species' global extents over long time periods (ca. 20-40 years) (Gaston & Fuller, 2009; Hurlbert & Jetz, 2007) with minimum false presence rates at spatial grains of 100-500 km (Hurlbert & Jetz, 2007).

Updated text (Methods, Species distribution data):
Spatial biodiversity data coverage, hereafter referred to as data coverage, can be calculated at any spatial, temporal, or taxonomic resolution of interest. Doing so requires an estimation of expected biodiversity. The spatial resolution of analysis is primarily determined by the data used to estimate a species expected range. That is, higher spatial uncertainty in the species range expectations necessitates coarser resolutions of analysis in order to minimize false presences and thereby not hold nations accountable for collecting data in areas where a species does not occur. We estimated terrestrial vertebrate diversity based composites of single species distribution maps. Previous empirical studies have shown that such expert-based range maps minimize false presences at spatial resolutions of 100-500 km to approximate species' global extents over long time periods (ca. 10-50 years) (Gaston & Fuller, 2009; Hurlbert & Jetz, 2007; Jetz et al. 2012). Therefore, we estimated diversity using an equal-area grid with the finest spatial grain appropriate for expert-based species distribution maps (110km x 110km).

We demonstrated the potential to estimate species distributions at finer spatial resolutions (55 and 27.5 km) based on output from published species distribution models (Ellis-Soto et al. 2021) for two example species (Supplemental Fig. 3).

Additional text (Methods, Species Status Information Index (SSII):

We demonstrated the sensitivity of the SSII to a range of spatial resolutions (110, 55, and 27.5 km) for two example species based on published species distribution models (Ellis-Soto et al. 2021) and found that SSII values decrease predictably at finer spatial resolutions (Supplementary Fig. 3, Supplementary Text).

Supplementary results text:
We demonstrated the potential of the SSII to be calculated across a range of spatial resolutions (110, 55, and 27.5 km) for two example species (Supplemental Fig. 3). Because expert-based species distribution maps are not accurate at finer spatial resolutions, we used thresholded output from published species distribution models to estimate ranges at finer resolutions for two hummingbird species, the Glowing puffleg (*Eriocnemis vestita*) and White-sided hillstar (*Oreotrochilus leucopleurus*) (Ellis-Soto et al. 2021). Thresholded species distribution model outputs were rescaled to three equal-area grids (110, 55, and 27.5 km) and intersected with records collected over the previous two decades (2000-2019) (Supplemental Fig. 3 a,b). We computed annual SSII values over the same time period for both species. Unsurprisingly, data coverage decreased at finer spatial resolutions (Supplemental Fig. 3c). We compared SSII values based on each spatial resolution and estimated the slope of relationship for both species independently using linear regression (Supplemental Fig. 3d). For each comparison, we found that 95% confidence intervals of regression slopes overlapped between species, suggesting that relative SSII values scale consistently between resolutions among species. Therefore, while spatial scale clearly impacts the absolute value of the SSII, comparisons across species and regions are consistent across spatial resolutions.

2) I kind of see why the ideal distribution is equal sampling among grid cells. But then this opens up a lot of questions about EOO and AOO, and how the SSII performs in relation to these. The assumption that grid cells are empty then the species doesn't exist there, is not great, and potentially a fundamental flaw as the manuscript currently stands. But I understand you don't get 'absence' information. I think future work would be able to improve these indices by inferring absences based on number of records etc. - especially at large spatial scales. The authors mention this limitation towards the end of the manuscript. But, I think if the authors can demonstrate that this isn't a big issue. Maybe with the jaguar and peccary. Then this would really strengthen the manuscript. It would also be informative to know when this method works for some species as opposed to others

It seems the reviewer's main concern is about the potential for false presences in the range expectation (the discrepancy between AOO and EOO) that would result in a missing observation for that cell falsely declared as data absence. We address this issue by using a coarse enough grain size to be able to assume negligble false presences in the EOO and thus EOO = AOO. We have added additional text (listed in response to the previous comment) which we hope clarifies this point.

3) What about migrants? This is mostly related to birds. But as I understand, you take all records regardless of time of year and integrate them spatially with grids. But, this could influence the country's reporting as their 'range' only applies to a certain time of year. E.g., neotropical migrants. In the methods the authors argue that by taking the average over many species, some idiosyncrasies among species likely are minimal. But it would be really worth showing this empirically, instead of just making the conceptual argument. Perhaps you could use migrants as a case study.

We have focused our analyses on breeding and resident ranges as opposed to migratory and wintering ranges. We expect every nation to have the burden of assessing distribution status for any breeding species annually, irrespective of how long that species may reside in the country. We have added text to clarify this point.

Additional text (Methods, Species distribution data):
For bird species, we restrict our analysis to breeding and resident ranges, excluding migration and wintering habitats. Our analysis therefore expects nations to assess distribution status for any breeding species annually, irrespective of the duration that the species resides within the country.

4) Data availability: According to the version of the manuscript I received, the data availability statement is as follows: "All data is available at the Map of Life (www.mol.org).". I'm a big fan of MOL, but I don't know how to get data our of there. I don't see any type of 'download data' button or tabs. Do I need an account? I just spent about 20 minutes trying to download some datasets, but didn't get anywhere. Making an account is prohibitive, and given the push for open access data, this shouldn't be a necessity. I even tried tracking down the GitHub repo (https://github.com/MapofLife/indicators), but didn't see any actual data here. Given the requirement to publish in PLoS of all data underlying the findings being "fully available", I would hope that the authors can make these data available properly. Even if you can actually get them from MOL and I'm just dense in my ability to do so, I still think as a stand alone paper these data should be available so that the results and findings are reproducible. That is, the data from 1950-2019 for each species, and the SSII and SSEI for each species, as well as probably separate datasets for the country-level averages of these, should be made available in a repository that is permanently archived before acceptance of this manuscript.

We are honored by the enthusiasm for Map of Life. All annual national SSII and SSEI for each of the studied taxa will of course be fully publicly available in MOL upon publication of the study. We have recently launched a new user interface at MOL (still in test phase) that enables direct download of all reshareable datasets, which includes the expert range maps for birds (e.g. https://mol.org/species/map/Tockus_deckeni) . We'd be thrilled to receive (happily anonymous) feedback on how we could further improve this functionality (https://docs.google.com/forms/d/e/1FAIpQLSd1DJkQ_5s3rHuIa7pNzc4Hd015C37vTMio2ztqufv8mUBXBA/viewform). Unfortunately, for several range map datasets (e.g. those developed by Red List assessors and held by IUCN) we do not have the permission to reshare, but applications for accessing these data can be made to these providers directly.

We have now changed the MS text as follows: "All indicator results are directly accessible for download at mol.org/indicators. All underlying data are visually accessible on the species pages of mol.org and where not directly downloadable there available from the sources listed."

Minor comments:
If PLoS allows it, I would suggest using some subheadings in the main text. There are a few jumps to new paragraphs where the flow is not great, and I think subheadings could help alleviate this.

Thank you, we have added subheadings which we hope are helpful to the reader.

Lines 60-61: Totally agree!

Line 69-74: I don't doubt these statements at all. I'm just wondering if there is any references/qualitative research surrounding this? Mainly, because if there is, it would be cool to highlight it. I suspect such references would be hard to come by, however. But perhaps at the very least, the authors could provide an example of a few nations where they incentivized an improved information base and this was led from a national planning coordination? This is discussed a little bit when the results are interpreted, but an example here would help sell this important point a bit more.

We have added the following example.

Additional text:
For example, the activities of the Mexican National Commission on Biodiversity (CONABIO), a permanent commission of the Mexican federal government, have led to strongly increased biodiversity information in that country that supports conservation decisions in the region (Sarukhán et al., 2015).

Line 81: "advance and globally implement". So is this method used previously? Has it been published before? Or is what you are doing now, the first time it is being described? This is related to my main point above. If it was published through geo bon, to what extent?

The SSII has not been published previously. However, a preliminary version of the metric is available online at mol.org. We have updated the text to clarify this point.

Original text:
Specifically, we advance and globally implement the Map of Life Species Status Information Index (SSII) which was developed under the auspices of the GEO Biodiversity Observation Network…

Updated text:
Specifically, we present and globally implement the Map of Life Species Status Information Index (SSII) which was developed under the auspices of the GEO Biodiversity Observation Network….

Lines 99-100: Here you say, "not just by the number of records, but also by how effectively the records cover a species' full geographic range". But, then what is the actual difference between SSII and SSEI in words? Or is SSEI just a component of the SSII? Perhaps removing this word 'effectively' and rephrasing will help show that there is indeed a difference between SSII and SSEI?

Thank you for highlighting this sentence, we have updated the text to improve clarity.

Original text:
For a given species, SSII is determined not just by the number of records, but also by how effectively the records cover a species' full geographic range.

Updated text:

For a given species, SSII quantifies the proportion of the range with data, but not how effectively this data is distributed across the proportion of the range it covers.

Line 138: This, to me, is the most likely scenario for Western Europe, South Africa, and Australia. Surely they haven't slowed in their coverage progress, necessarily? But just they are operating at maximum ability?

We agree with this point and have updated the text to reflect so.

Original text:
However, we anticipate that even under constant effort, nations' coverage may asymptote as marginal gains become more challenging to achieve

Updated text:
Therefore, asymptoting trajectories in data coverage may suggest that nations are operating at maximum capacity.

Lines 140-142: This is interesting! And I agree. But incredibly political. If I understand correctly, the authors are highlighting that, for example, Australia should begin to work on capacity-building in the Pacific Islands and/or Southeast Asia? This is way easier said than done, but I get the point by the authors.

Line 162: It is funny to think of this reference as 'outdated', but they downloaded data from GBIF in 2016, and only used 649 million occurrences (< half of what is in GBIF now). I would bet that the proportion of data from museum specimens has drastically decreased by now in 2020, mainly due to programs like iSPOT and iNaturalist growing in popularity and being integrated into GBIF.

We agree that is likely true for more recent data collection and have added additional text to reflect this point.

Original text:
Nearly all records for birds in GBIF (>90%) come from direct observations, as opposed to museum specimens which constitute the primary source of records for amphibians, reptiles, and to a lesser degree, mammals (Troudet et al. 2017).

Updated text:
As of 2016, nearly all records for birds in GBIF (>90%) came from direct observations, as opposed to museum specimens which constitute the primary source of records for amphibians, reptiles, and to a lesser degree, mammals (Troudet et al. 2017). However, these differences in sources are likely to narrow as citizen scientist programs not restricted to birds continue to grow in popularity (e.g. iNaturalist).

Lines 178-181: I think this is great and I agree!

Lines 203-207: Interesting!!

Line 565: Are these range maps available somewhere in a repository? Can they all be downloaded from MOL for instance? This is core to your analysis, so it would be good to highlight this, although I wouldn't suggest you have to make these available.

As referenced in the text, range maps for amphibians and mammals are available from IUCN. Range maps for reptiles are available from the Global Assessment of Reptile Distributions. We will make the bird ranges available for download through the data availability statement.

Line 578: What about duplicates in GBIF? There are a lot, often in GBIF. https://recology.info/2016/03/scrubr/

Duplicate records would not influence the SSII, as coverage is determined based on the proportion of grid cells with data (regardless of amount), but would influence the SSEI results. We have thus removed duplicate records and added the following text to the Methods:

Additional text:
Duplicate records with the same species name, coordinates, and date were removed.

Line 617-624: See general comments above.

See response to general comment above.

Line 626-642: ++1 for using geohashes. The field of ecology rarely uses these! Some information here about where your analyses were performed would be handy. Was it using BigQuery (I'm guessing)? Or qGIS?

We have added the following text to clarify the methods used.

Original text:
…. Geohash level 5 bounding boxes and the respective proportion contained within each geometry were generated for national boundaries, species ranges, and the equal-area grid. All three datasets were then intersected based on common geohashes. ….

Updated text:
….Geohash level 5 represents geographic space with 5 km bounding boxes at the equator that increase in precision as they approach the poles. Geohash level 5 bounding boxes and the respective proportion contained within each geometry were generated for national boundaries, species ranges, and the equal-area grid using the python-geohash library. Spatiotemporal occurrence records were encoded with geohash level 5 using the R package “geohashTools” (Chirico 2020). All three datasets were then intersected based on common geohashes in R 4.0.0 (R Core Team, 2020). …

Lines 712-715: I am not fully following this part. Could you elaborate please.

We have added text to clarify this point.

Original text:

Restricting class-wide SSII values for each class to the years with comparable number of records (58k-118k records; i.e., years 1950s for birds, 1990s to mid 2000s for mammals, and late 2010s for the other two classes), mean SSII was highest for amphibians (0.026), reptiles (0.017), mammals (0.009), and lowest for birds (0.005) (Fig. 3d).

Updated text:
We compared SSII values among taxa when a comparable number of records were collected (i.e. for a given number of records, how did SSII differ among taxa?). Restricting class-wide SSII values for each class to the years with comparable number of records (58k-118k records; i.e., years 1950s for birds, 1990s to mid 2000s for mammals, and late 2010s for the other two classes), mean SSII was highest for amphibians (0.026), reptiles (0.017), mammals (0.009), and lowest for birds (0.005) (Fig. 3d).

Lines 830-849: Presumably these will be made available in csv format or some other format for readers? Also, see comments on data availability above.

Yes, we are happy to provide these in a csv for download.

Reviewer #2:

I enjoyed the introduction it contained a lot of good relevant background information that will be easy to digest for non-experts.

Given the dearth of data at a global scale it is very difficult to identify indicators that will be meaningful a large scale. The SSII and SSEI show promise. SSII quantifies spatiotemporal data while SSEI adds an adjustment for sampling effectiveness. A strength of SSII is indicated by the best sampled group where birds SSII has increased but sampling effectiveness has decreased due to the redundant sampling of citizen science efforts.

Most of the discussion is about the avian results which makes sense due to the higher level of data available for birds. It would be useful to have more text about other taxa. From figure 4 there is less applicability for non-bird data. Is it useful at all for those taxa, specifically reptiles and amphibians?

We do feel these metrics are useful for other taxa, particularly for highlighting how taxonomic biases continue to persist. By quantifying data coverage and sampling effectiveness, we feel these metrics are critical identifying species and locations which are most critically understudied, which is particularly important for other taxa. Our hope is that these metrics help inform how to best leverage existing resources and track progress of these efforts for all taxa. We have added text relating to other other taxa.

Additional text:
Although data collection in terms of number of records for mammal species consistently outpaced that for amphibians and reptiles, data coverage for mammals was lowest in recent years (Fig. 3c).

This suggests that despite increasing data availability for all taxa, a majority of nations are not making progress in closing information gaps for mammals, amphibians, and reptiles.

The limitations section is appreciated. To strengthen your argument for use of these metrics, I would like to hear clear recommendations from the authors to make the metrics more valuable. There clearly is not sufficient data for global recommendations to track some specific taxa to give global coverage, maybe someday. It appears that focused, periodic monitoring of endemic taxa would be the best use of resources, especially at the national level. Since data lack for most groups, can the authors make any recommendations of a subset of taxa that would be useful for nations to track?

We appreciated this comment and concur with the notion that focusing on a subset of species for monitoring will be necessary. We are hesitant, however, to give specific recommendations for what essentially represents a triage in this paper - it simply does not directly flow from our specific analyses and results. Given the importance of getting this right we feel this reflection might be best left to a paper dedicated to this topic. We do offer a monitoring recommendation in the following, Conclusions section: "Our findings suggest that trends in data coverage fundamentally differ by taxa and region and highlight the need to complement and reassess biodiversity sampling strategies to most effectively translate data collection into biodiversity knowledge useful for management and decision-making."

Fig 1b: It is unclear to me where the numerators of the aggregate country cover calculations come from.

We have added additional information in the figure caption to help clarify this point.

Original text:
At the species level, the SSII is given as the proportion of cells expected occupied with records in a given year. Species level SSII can be aggregated to the national level via two formulations. National SSII for a given taxonomic group takes the mean coverage across all species expected in a country, Steward's SSII adjusts the mean coverage across species by their respective national stewardship.

Updated text:
At the species level, the SSII is given as the proportion of cells expected occupied with records in a given year. In this hypothetical example, coverage is 0.83 and 0.67 for species where 5 out of 6 and 2 out 3 expected grid cells have data. Steward's SSII adjusts this coverage by their respective national stewardship (0.83 and 0.2). Species level SSII can be aggregated to the national level via two formulations. National SSII for a given taxonomic group takes the mean coverage across all species expected in a country (0.75). Steward's SSII adjusts the mean coverage across species by their respective national stewardship (0.8).

Starting at line 121: This starts out as a discussion of birds but it is unclear from the second half of the paragraph if it is still just birds or all data in the figures. This needs clarification and separate paragraphs to help the reader.

We have separated these paragraphs to help clarify this discussion.

Line 130: clarify does the 42% refer only to birds. Please add birds to clarify.

We have added text to clarify that this refers to average coverage across taxa.

Lines 134-136: which figure does this refer too? Any comments on the non-avian portion of the figure?

We have added a reference to the relevant figure.

Figure 5 legend error? Quadrant definitions 1 and 3 as well as 2 and 4 are identical. It is correct in the text, line 185. Please consider changing the color of Fig 5a: light blue for background for quadrant is confusing. It printed out nearly identical blues in my printout.

We have updated the figure legend as follows:

Original text:
The four colored quadrants represent nations with: (1) coverage below the global mean and no or decreasing trend; (2) coverage below the global mean and an increasing trend; (3) coverage below the global mean and no or decreasing trend; (4) coverage below the global mean and an increasing trend.

Updated text:

We categorized nations into the following four main types based on Steward's SSII status and trends over the previous decade: (1) coverage ***less than*** the global mean with ***no or decreasing*** trend (2010-2019) (42% of nations); (2) coverage ***less than*** the global mean with an ***increasing*** trend (25%); (3) coverage ***greater than*** the global mean with ***no or decreasing*** trend (16%); (4) coverage ***greater than*** the global mean an ***increasing*** trend (17%).

Line 192: I am not convinced by the discussions correlating trajectories with political decisions and national infrastructures changes.

We have revised this section.
Original text:
Political decisions and national infrastructure may have a large influence on data coverage trajectories. For example, the relaxation of stringent data sharing restrictions on biodiversity research in Brazil in 2015 (Escobar, 2015) appears to have aided in a significant uptick in data coverage. Governmental support for and establishment of a national biodiversity program (CONABIO) (Sarukhán et al., 2015) has placed Mexico on a strong, and increasing, trajectory in both the 20th and 21st centuries. South Africa has achieved particularly high coverage of its biodiversity due to both long-term national policies and large-scale atlasing efforts, such as the Southern African Bird Atlas Project (Cherry, 2005; Harrison et al., 2008).

Updated text:
Biodiversity data coverage within Mexico has followed a strong, and increasing, trajectory in both the 20th and 21st centuries. Despite lower coverage through periods of the 20th century, South Africa has had similarly strong and increasing data coverage over the previous decade. Many nations which had historically limited data coverage showed recent increases in coverage, for example Brazil. These trajectories in data coverage may be due to political decisions and national infrastructure which supports biodiversity data collection and mobilization. For example, the establishment of a national biodiversity program (CONABIO) (Sarukhan et al. 2015) and large-scale atlasing efforts, such as the Southern African Bird Atlas Project (Cherry et al. 2005; Harrison et al. 2008).

Paragraph 201 is strong and will be useful for the community to hear.

Would a sliding window view of the data be feasible? The decade level analysis is necessary due to data but would the sliding window show trends better?

We are uncertain of the reviewer's meaning here and/or the advantage a sliding window or moving average would provide in this context.

Reviewer #3:
[identifies himself as Dominique G Roche]

I was pleased to review PBIOLOGY-D-20-03303_R1 "Global and national trends in documenting and monitoring species distributions". Overall, I think that the research has considerable value. Reducing biodiversity information gaps is a global priority and the proposed framework appears sound. That being said, I am no expert in biodiversity conservation and the relevance/novelty of the indices proposed should be assessed by reviewers with greater expertise in this field. My main criticism of the manuscript is that the text and some of the concepts were difficult to understand at times. Many sentences would benefit from being more clearly written and the figure/table captions were often confusing and/or lacked sufficient information. The figures are critical for readers to understand the content of this manuscript - greater effort should be invested in ensuring that the captions clearly walk the readers through the results.

It would be helpful if the authors included a glossary as a text box to prominently define/explain key terms such as GBIF, MOL, Global/National/Stewards' SSI, SSEI. I think this addition would greatly enhance the ease with which the paper could be read and its accessibility to a broad readership.

We have added a glossary of these terms to Figure 1, if the editor allows for it within this format.

The authors mention the importance of scale in the introduction (L64) but there is no justification for the grid size of 110 x 110 km used in the study other than it was "the finest spatial grain appropriate" (L569). There is also no discussion of the potential consequences of using a different scale in their analysis.

We have provided empirical examples of how the SSII can be extended to finer spatial resolutions for two example species (Supplementary Fig. 3). We have added text throughout the manuscript to clarify the use of the current spatial resolution and implications for extending to finer resolutions.

Additional text (Main text):
Our methodology and analysis infrastructure is capable of flexibly accommodating different spatial resolutions (Supplementary Fig. 3, Supplementary Text) as more accurate information on species' ranges becomes available (i.e. through species distribution modeling) for a broader range of taxa.

Original text (Methods, Species distribution data):
Spatial biodiversity data coverage, hereafter referred to as data coverage, can be calculated at any spatial, temporal, or taxonomic resolution of interest. Doing so requires an estimation of expected biodiversity. We estimated terrestrial vertebrate diversity based on composites of single species distribution maps, which have been shown to approximate species' global extents over long time periods (ca. 20-40 years) (Gaston & Fuller, 2009; Hurlbert & Jetz, 2007) with minimum false presence rates at spatial grains of 100-500 km (Hurlbert & Jetz, 2007).

Updated text (Methods, Species distribution data):

Spatial biodiversity data coverage, hereafter referred to as data coverage, can be calculated at any spatial, temporal, or taxonomic resolution of interest. Doing so requires an estimation of expected biodiversity. The spatial resolution of analysis is primarily determined by the data used to estimate a species' expected range. That is, higher spatial uncertainty in the species range expectations necessitates coarser resolutions of analysis in order to minimize false presences and thereby not set up a data collection expectation for areas where a species does not occur. We estimated terrestrial vertebrate diversity based composites of single species distribution maps. Previous empirical studies have shown that such expert-based range maps minimize false presences at spatial resolutions of 100-500 km to approximate species' global extents over long time periods (ca. 10-50 years) (Gaston & Fuller, 2009; Hurlbert & Jetz, 2007; Jetz et al. 2012). Therefore, we estimated diversity using an equal-area grid with the finest spatial grain appropriate for expert-based species distribution maps (110km x 110km).

We demonstrated the potential to estimate species distributions at finer spatial resolutions (55 and 27.5 km) based on output from published species distribution models (Ellis-Soto et al. 2021) with high accuracy validated at finer-resolution for two example species (Supplemental Fig. 3).

Additional text (Methods, Species Status Information Index (SSII):
We demonstrated the sensitivity of the SSII to a range of spatial resolutions (110, 55, and 27.5 km) for two example species based on published species distribution models (Ellis-Soto et al. 2021) and found that SSII values decrease predictably at finer spatial resolutions (Supplementary Fig. 3, Supplementary Text).

Supplementary results text (figure below):
We demonstrated the potential of the SSII to be calculated across a range of spatial resolutions (110, 55, and 27.5 km) for two example species (Supplemental Fig. 3). Because expert-based species distribution maps are not accurate at finer spatial resolutions, we used thresholded output from published species distribution models to estimate ranges at finer resolutions for two hummingbird species, the Glowing puffleg (*Eriocnemis vestita*) and White-sided hillstar (*Oreotrochilus leucopleurus*) (Ellis-Soto et al. 2021). Thresholded species distribution model outputs were rescaled to three equal-area grids (110, 55, and 27.5 km) and intersected with records collected over the previous two decades (2000-2019) (Supplemental Fig. 3 a,b). We computed annual SSII values over the same time period for both species. Unsurprisingly, data coverage decreased at finer spatial resolutions (Supplemental Fig. 3c). We compared SSII values based on each spatial resolution and estimated the slope of relationship for both species independently using linear regression (Supplemental Fig. 3d). For each comparison, we found that 95% confidence intervals of regression slopes overlapped between species, suggesting that SSII values scale consistently between resolutions among species. Therefore, while spatial scale clearly impacts the absolute value of the SSII, comparisons across species and regions should largely be consistent across spatial resolutions.

The explanation of how 'expected occupied cells' were determined is limited (L587-590).

We have added the following text in the description of species distribution data:

Additional text (Methods, Species distribution data):
The grid cells intersecting a species range map were considered to be expected occupied by that species, and thus served as the baseline against which existing records were compared to.

Few of the test statistics mentioned in the methods (L683-688) are reported in the manuscript. P-values alone are often presented (L722-769) or relationships and differences are only referred to as 'significant' or 'non-significant'.

We are happy to provide more information on test statistics. However, we are uncertain which results the reviewer is referring to. If the reviewer could provide more details, we would be happy to provide them. In addition, the previous reference to ANOVA was a typo from a previous version of the text and has been removed.

The manuscript lacks a data availability statement. It is not sufficient to state (in the metadata) that the data are "available at the Map of Life (www.mol.org)". The exact link to the specific data used in the study should be provided for reproducibility purposes. Ideally, the data would be downloaded from mol.org and archived in a trusted repository, unless the authors can demonstrate that MOL is funded for the next 50 years (as is the case for trusted repositories). This is because the data disappear is MOL is no longer supported. If the data are available via the GBIF, the authors could share the script used to access them via the API.

I would like to see the authors share the R script used in this study (e.g., via the OSF, Figshare, Zenodo, or some other trusted repository). Given that the aim of the study is to reduce information gaps, it would be nice to see the authors lead by example and readily share their data and code.

See also response to reviewer 1. MOL has submitted an application to be formally recognized as a trusted data repository. If that status is not in place by the time of publication, all information necessary to replicate the results will in addition to on MOL also be made available on a formally already recognized repository.

Minor comments:

L21. I suggest rephrasing this sentence as: "Here, we propose novel indicators of biodiversity data coverage and sampling effectiveness, and analyze national trajectories in closing spatiotemporal knowledge gaps for terrestrial vertebrates (1950-2019)."

Thank you, we have changed the statement on L21.

L27. I suggest rephrasing this sentence as: "However, we found that a nation's coverage was stronger for species for which it holds greater stewardship."

Thank you, we have adopted this rephrasing on L27.

L40. Missing reference

We have added the appropriate reference.

L45-47. Grammar

We have changed this sentence to the following:

The status and trends of species' geographic distributions are directly related to species' ecological relevance, population size, and extinction risk, and are thus central to the conservation and management of species and their ecological functions.

L64. It is unclear what you mean by "expectation". This only becomes evident later in the manuscript.

We have updated the text to clarify this point.

Original text:
Indeed, prior work has revealed significant taxonomic and geographic gaps in the existing data and highlighted the importance of accounting for expectation and scale in the assessment of coverage.

Updated text:
Indeed, prior work has revealed significant taxonomic and geographic gaps in the existing data and highlighted the importance of accounting for expected diversity and scale sensitivity in data coverage assessments.

L67. Grammar. Key correlates of what? This sentence in unclear.

Thank you, we have updated the text to clarify.

Original text:
Scientists have identified a range of socio-economic and ecological drivers for gaps and biases in the current data and identified geographical access, availability of local funding resources, and participation in data-sharing networks as key correlates.

Updated text:
Scientists have identified a range of socio-economic, linguistic, and ecological drivers for gaps and biases in the current data and identified geographical access, availability of local funding resources, and participation in data-sharing networks as key correlates of data gaps

L71-72. This sentence is unclear… do you mean "allow changes in biodiversity data coverage over time to inform decision-making"?

Thank you for the suggestion, we have updated the text accordingly.

Original text:
Developing such an evidence base requires metrics that relate changes in biodiversity data coverage over time to decision-making.

Updated text:
Developing such an evidence base requires metrics that allow changes in biodiversity data coverage over time to inform decision-making.

L73. "nations […] stand to gain the greatest benefits from broadly improved biodiversity information" - why? It's unclear to me that this is the case for all nations.

Our point here is meant to justify nations as a unit of analysis, due to the fact that responsibility for reporting coordinating management ultimately falls to national governments. We have updated the text to reflect this.

Original text:
As stewards of their biodiversity, nations hold the key to incentivizing an improved information base and stand to gain the greatest benefits from broadly improved biodiversity information by enabling monitoring and robust management decisions.

Updated text:
As stewards of their biodiversity and political unit responsible for coordination, nations hold the key to incentivizing an improved information base and stand to gain the greatest benefits from broadly improved biodiversity information by enabling monitoring and robust management decisions.

L76-77. Can you cite one or multiple sources in support of this statement? What about the references cited on L229?

Thank you, we have added these references here.

L79-81. This sentence is difficult to read. I suggest removing "an updatable framework and". Perhaps write a second sentence after this first one explaining what you mean by 'updatable framework'.

We have updated the text to improve clarity.

Original text:
Here, we provide an updatable framework and two national indicators in support of the global assessment, monitoring, and decision-support around annual trends in spatiotemporal biodiversity information.

Updated text:
Here, we provide two national indicators in support of the global assessment, monitoring, and decision-support around annual trends in spatiotemporal biodiversity information. These metrics are integrated within a flexible, updatable analytical framework.

L88-90. I suggest modifying this sentence as follows: "We provide a first global assessment of… [expand] … for terrestrial vertebrates as well as infrastructure to continue tracking these indices at Map of Life (https://mol.org/indicators/coverage)."

We have expanded the text as suggested.

Original text:
We provide a first global assessment for terrestrial vertebrates in this study and infrastructure to continuously track these indices into the future at Map of Life (https://mol.org/indicators/coverage).

Updated text:
We provide a first global assessment for trends in data coverage and sampling effectiveness for terrestrial vertebrates as well as infrastructure to continuously track these indices into the future at Map of Life (https://mol.org/indicators/coverage).

L95. Grammar

We have addressed the grammatical issues as follows:

Original text:
National SSII restricts this calculation to the range cells inside a particular country.

Updated text:
The National SSII is calculated using the same method as the Global SSII, but is restricted to the range cells inside a particular country.

L93-97. I suggest directing the reader to the relevant panel(s) of Fig. 1 after each term is explained. The current explanation of Steward's SSI is fairly limited in my opinion. If you cannot expand on it in the main text due to the word limit, I would direct the reader to the methods by adding "(see Methods") here rather than in the following sentence.

We have added figure references throughout this paragraph.

L102. Is the 'ideal uniform distribution based on Shannon's entropy' really the optimal sampling strategy? Again, I'm no expert in this field but I would have liked to see the rational for this choice, perhaps in the methods.

We have added additional text to clarify this point.

Additional text (Methods, SSEI):
The SSII quantifies the proportion of a species' range with data. The SSEI quantifies how evenly this data is distributed among the grid cells it covers. As formulated, the SSEI metrics penalizes uneven sampling based on the size of the discrepancy of the number of records contained within sampled grid cells (Supplementary Fig. 4). For example, low values of SSEI correspond to situations where a small proportion of grid cells contain many duplicate records and the remaining grid cells contain very few or a single record. In this case we consider ideal sampling to be entirely uniform distribution of records because uneven sampling suggested geographic biases in data collection. The SSEI therefore quantifies the degree of geographic biases within the portion of the range that is sampled.

L110-112. This sentence in unclear.

We updated the text as follows to improve clarity.

Original text:

Global SSII was consistently higher for the peccary than the jaguar in the first decade, but the difference was much smaller than the substantial 2- to 10-fold higher annual number of peccary records.

Updated text:
The number of records collected for the peccary annually was substantially higher than for the jaguar, ranging from 2- to 10-fold higher data collection. Subsequently, Global SSII was consistently higher for the peccary than the jaguar, but the difference in values was narrower than the difference in data collection would suggest.

L108-119. Please refer the reader to specific panels in Fig. 1 throughout this paragraph.

We have added text references to all panels.

L121. I suggest using a word other than 'exploded'.

We have replaced ‘exploded’ with ‘proliferated’.

L134. This sentence should be supported by a reference to a figure or paper.

We have added a reference to Fig. 4e.

L 143. Ref needed.

We have added a reference to Fig. 4e.

L144-147. The rational for this statement needs to be better explained.

We have updated the text as follows:

Original text:
Our results additionally highlight the importance of regionally targeted capacity-building and data mobilization initiatives such as GBIF’s Biodiversity Information Fund for Asia and Biodiversity Information for Development program focused in sub-Saharan Africa, the Caribbean, and the Pacific.

Updated text:
Our results underscore the importance of regionally targeted capacity-building and data mobilization initiatives which support regions with historically limited data coverage. Such efforts include GBIF’s Biodiversity Information Fund for Asia and Biodiversity Information for Development program focused in sub-Saharan Africa, the Caribbean, and the Pacific.

L149-150. This sentence is unclear.

We have updated the text as follows:

Original text:
While immense in magnitude and potentially useful for other ecological applications, not all data has translated to new knowledge of species distributions.

Updated text:
Despite the astounding accumulation of biodiversity records, not all data has translated to new knowledge of species distributions. While potentially useful for other ecological applications,

L155. Unclear.

We have updated the text as follows:

Original text:
National trends in effectiveness also appear to be largely driven by the trends in sampling effectiveness for bird species, which has declined rapidly over the past two decades, constraining the direct conversion of the multitudinous accumulation of records into data coverage.

Updated text:
National trends in effectiveness also appear to be largely driven by the trends in sampling effectiveness for bird species, which has declined rapidly over the past two decades, constraining the direct conversion of the immense accumulation of data into data coverage.

L163. What is meant by "for the same number of records". Can you point the reader to a figure?

We have added a reference to Fig. 3d.

L170. Ref to figure needed.

We have added a reference to Fig. 3e.

L175. For clarity, please expand (perhaps in parenthesis) on what you mean by "effective and complementary".

We have added the following parenthetical: “(i.e. addresses undersampled species or regions)”

L180. Given the importance of MOL in the context of this study, it would be helpful to have a box clearly explaining what it is.

We have added a glossary of terms to Figure 1.

L185-188. This is repeated twice, on L690-694 and L754-758. I have a really hard time making sense of Fig 5a based on this description and the figure caption…

We have updated the text as follows:

Original text:
We categorized nations into the following four main types based on Steward's SSII status and trends over the previous decade: (1) coverage below the global mean and no or decreasing trend (42% of nations);

(2) increasing coverage, but below the global mean (25%); (3) coverage above the global mean, but no or decreasing trend (16%); (4) both coverage above the global mean and an increasing trend (17%).

Updated text:
We categorized nations into the following four main types based on Steward's SSII status and trends over the previous decade: (1) coverage ***less than*** the global mean with ***no or decreasing*** trend (2010-2019) (42% of nations); (2) coverage ***less than*** the global mean with an ***increasing*** trend (25%); (3) coverage ***greater than*** the global mean with ***no or decreasing*** trend (16%); (4) coverage ***greater than*** the global mean an ***increasing*** trend (17%).

L232-233. Grammar

We have updated the text as follows:

Original text:
However, results can vary dramatically with specific methodology and structure of input data, resulting in competing recommendations for their development and use.

Updated text:
However, richness estimates from extrapolation approaches can vary dramatically with the specific methodology used and structure of input data. As such, there are competing recommendations for their development and use.

L234. What are "limited transparencies of extrapolation approaches"? This sentence would benefit from being shortened. Perhaps split it into two?

We have updated the text as follows:

Original text:
The SSII avoids potential pitfalls and limited transparencies of extrapolation approaches by relating record collection directly to best-possible species-level expectations, allowing for decision support at the species level.

Updated text:
The SSII avoids potential pitfalls and the limited transparency of extrapolation approaches by relating record collection directly to best-possible species-level expectations. Therefore, the SSII allows for decision support at the species level which is not possible with extrapolation approaches.

I hope my comments are helpful.
Best regards,
Dom Roche

Reviewer #4:
[identifies himself as Jonathan Lenoir]

General comments

I read the work from Ruth Y. Oliver et al. with great interest and I think the authors are tackling an important topic which echoes the recent literature highlighting gaps and biases in our knowledge of global biodiversity distribution (and redistribution). In this manuscript, the authors focused on terrestrial vertebrates to assess both the global and national trends in the sampling effort to collect reliable data on species distribution. By doing so, the authors aim at highlighting, for each country separately, spatiotemporal trajectories in closing knowledge gaps in the distribution of terrestrial vertebrates. To achieve this goal, the authors built two species-specific metrics, namely (i) the species status information index (SSII) and (ii) the species sampling effectiveness index (SSEI), while accounting for country-specific stewardship of species (i.e. how responsible a country is for a given species, which is determined by the portion of the focal species global range that is occurring in the focal country) when aggregating across species the information per country. Using these two metrics, the authors found a rapid global increase, during the last decades, in the relative sampling coverage of each species distribution (SSII), especially so for some taxonomic groups, like birds. However, this rapid global increase in SSII further amplified the strong and existing geographic bias among countries, leading to country-specific SSII trajectories. Noteworthy, the authors suggested that the tremendous growth of species occurrence records in some countries failed to directly translate into newfound knowledge due to a sharp decline in sampling effectiveness (SSEI).

The manuscript is very well written and the figures are really helpful and informative, not only for displaying the main findings (Figs. 3-5) but also for helping the reader to understand the metrics used by the authors (especially Figs. 1 and 2). I really liked it as these figures are very intuitive, thus I would like to congratulate the authors for this effort. This said, I do have several important concerns and reservations that, I think, warrant publication of the manuscript as is. My first and main concern is about the SSEI metric which I think is somewhat flawed and inherently related to SSII. Indeed, the SSEI metric works such that the more occurrence records, the more likely it is that the distribution is uneven among the sampled grid cells. Let's for instance take the most extreme case of one single occurrence record for a given species. This means that only one grid cell among all the possible grid cells that are expected to be occupied is actually occupied and thus SSII is very low for that species, either globally or at the national level (except if the focal country is of the size of the occupied grid cell). For the SSEI metric, then the value is 1 just because the distribution is completely even across the occupied cells (1 occurrence record occurring in one grid cell: perfectly even). Hence, there is a mathematical relationship between SSEI and SSII, which are not fully independent from each other, such that the correlation can only be negative and the data constrained within an upper triangle when relating SSII (x-axis) against SSEI (y-axis). This is very well illustrated by the inset plot in Fig. 4d. As it is now, in my opinion, the SSEI metric is a bit useless, except towards very large values of SSII (when reaching completeness in the species distribution), because only then the SSEI metric bring

new insights. However, for low SSII values, the SSEI has no meaning. A more meaningful and useful, I think, version of the SSEI
metric should account for the total number of empty cells to further penalize the SSEI metric when only a few cells are occupied among the number of expected cells supposed to be occupied (cf. low SSII values).

We appreciate the reviewer’s interest in the SSEI and the reflections offered. We have developed and considered the SSEI (sampling effectiveness) and SSII (data coverage) metrics very carefully. We do not share the concern that they are redundant. The SSII is the proportion of a species’ expected range with records (# of cells with records/# of expected cells). The SSEI quantifies the evenness of the distribution of records within sampled grid cells based on the entropy of the realized distribution of records normalized by the entropy of the ideal distribution of records (in this case equal number of records within sampled grid cells).

Indeed, for a given number of samples and a given value of the SSII, the SSEI may vary independently from its minimum value to 1. The minimum value of the SSEI is dependent on the total number of samples (see Supp. Fig 3), but does not really depend on the number of cells those samples are distributed over or how many cells possibly exist (i.e. including those with zeroes). Computing SSEI over all cells in the species range (including the zeroes where no samples were collected) as the reviewer suggests would also be valid and indeed we considered this version of the metric, but in this case the SSII and SSEI become more closely related and it becomes difficult to distinguish the qualities of different sampling patterns from the two measures. By focusing SSEI on the sampled cells, the two measures become more or less independent and thus describe the sampling along two separate axes: the coverage of the species range and the uniformity of sampling. The SSII simply quantifies the proportion of the species’ range with data. The SSEI quantifies how evenly this data is distributed among the grid cells it covers. Therefore we see value in presenting these two metrics in combination.

In response to the point that SSEI inherently decreases as the number of records increases, we have added panel (b) to Supplementary Figure 3 (copied below) which demonstrates the sensitivity of the SSEI to the number of records given constant coverage. As formulated, the SSEI metrics penalizes uneven sampling based on the size of the discrepancy of the number of records contained within sampled grid cells. In this figure we used simulated data where the sampled portion of the range contains varying discrepancies in the number of records in grid cells (i.e. grid cells either contain a single record vs. 2, 5, 10, 100, and 1000 records). From panel (a) we see that in the case where sampled grid cells either contain 1 or 2 records, SSEI is nearly maximized. In the case where sampled grid cells either contain 1 or 1000 records, SSEI varies over a much larger range, reaching a minimum when >95% of the sampled range contains a single record per grid cell and <5% of the range contains grid cells with a 1000 records per grid cell. Panel (b) displays the same data in terms of the total number of records. Again, SSII is constant across these situations. In panel (b) we see that in each scenario (level of discrepancy between number of records in grid cells), SSEI is in fact maximized at the greatest number of records because this corresponds to evenly distributed duplication of grid cells.

The reviewer highlights an important point about the situation in which a single record is collected. These cases are excluded from national averages of SSEI. We have added text in the Methods section to clarify this point.

We hope that this more detailed explanation is useful and have added text to the manuscript to clarify the SSEI to readers.

Original text (Main text):
Furthermore, the annual time units and a relatively coarse grid for the SSII patterns presented here are insensitive to spatiotemporally dense data that could reveal seasonal dynamics and additional insights offered by repeat samples (e.g., in occupancy modelling frameworks) (Mackenzie et al. 2002). Similarly, by penalizing uneven sampling, the SSEI ignores applications which require repeat sampling.

Updated text:
Furthermore, the annual time units and a relatively coarse grid for the SSII patterns presented here are insensitive to spatiotemporally dense data that could reveal seasonal dynamics and additional insights offered by repeat samples (e.g., in occupancy modelling frameworks) (Mackenzie et al. 2002). Similarly, by penalizing uneven sampling, the SSEI ignores applications which require repeat sampling.

Additional text (Methods, SSEI):
The SSII quantifies the proportion of a species' range with data. The SSEI quantifies how evenly this data is distributed among the grid cells it covers. As formulated, the SSEI metrics penalizes uneven sampling based on the size of the discrepancy of the number of records contained within sampled grid cells (Supplementary Fig. 4). For example, low values of SSEI correspond to situations where a small proportion of grid cells contain many duplicate records and the remaining grid cells contain very few or a single record. In this case we consider ideal sampling to be entirely uniform distribution of records because uneven sampling suggested geographic biases in data collection. The SSEI therefore quantifies the degree of geographic biases within the portion of the range that is sampled. Species without data or with data only within a single grid cell are excluded from national averages.

My second major concern is about the very strong assumption that terrestrial vertebrate species have completely static distribution throughout the 70 years of the study period (1950-2019) and that any species range shifts would have little impact on the authors' analyses (see lines 570-572). This is especially problematic for the last three decades (1990-2019), during which evidence of species range shifts as climate warms were exploding (see Lenoir et al. 2020). Hence, I suggest the authors to carefully consider that important and recognized fact (species redistribution as global climate warms) in the scientific literature (Parmesan et al. 2003; Chen et al. 2011; Lenoir& Svenning 2015; Pecl et al. 2017; Lenoir et al. 2020) and at the very minimum discuss the implication of trans-boundary shifts in species distribution (e.g. species range expansions into new countries), which should alter the authors finding. Even better, but this involves quite some work I have to admit, would be to account for species range shifts by adjusting each species range map by means of species distribution models (SDMs), for instance. Alternatively, one cheaper solution involving less work for the authors would be to split the analyses into two periods to distinguish a period during which the authors' assumption is likely to hold (cf. a 30-yr period prior to climate warming: 1950-1979) and a second 30-yr period (1990-2019) during which species range shifts may alter the authors' findings, thus requiring to at least discuss the potential implications of trans-boundary shifts during this second time period. As for the authors' defence, terrestrial vertebrates are among the taxonomic groups showing the slowest (most often non-significant but not always: e.g. significant latitudinal range shifts for reptiles) velocities in species latitudinal range shifts, as opposed to marine species (see Fig. 3a in Lenoir et al. 2020). This said, terrestrial vertebrate species showed

significant upslope range shifts (especially amphibians, but also birds and mammals: see Fig. 3b in Lenoir et al. 2020) during the last three decades. But I assume elevational range shifts will have relatively more minor impacts on trans-boundary shifts, except maybe for some highly mountainous countries like Bhutan, Nepal, Lesotho, Andorra, Chile or Switzerland where new species may arrive from the neighbouring lowland countries. Hence, I really urge the authors to at least discuss those important implications of trans-boundary shifts if they do not deem necessary (or if they think it is impossible) to account for species range shifts in their analyses, especially during the most recent (1990-2019) period.

We are aware, also from our own research on the topic, of the substantial range dynamics that species have already seen over the past seventy years and these will accelerate in the future. And we strongly agree with the reviewer's assertion that incorporating information on shifting distributions will be important for global biodiversity monitoring and assessment. As the reviewer states, transboundary shifts may occur and would have important implications for national monitoring efforts. The expert range map data we use provides is characteristic, i.e. captures maximum extent, for a ca. 10-50y time range (Jetz et al. 2012, others) and thus largely encompassing any geographic dynamics during that time frame. It may, however, miss select pre-1970 occurrences and some very recent range shifts. The relatively coarse analysis grain of 110 km grid cells may help alleviate some of this issue, in particular for the elevational shifts that the reviewer mentions which usually play out over relatively short distances. Unfortunately, as the reviewer states, temporally dynamic distribution information is not available globally to support directly tracking shifting species ranges. We agree that species distribution models could fill this gap, however generating reliable models for all terrestrial vertebrates remains a very active area of research and is out of scope of the current study. The indices and associated analytical framework we have developed are capable of supporting alternate estimates of species distributions as they become available, which could be dynamic. To demonstrate this potential, we have added an additional demonstration of how our methodology can incorporate SDMs (Supplemental Fig. 3). We are uncertain of the alternate approach suggested by the reviewer, as both time windows would rely on the same, static representation of species ranges, but are happy to discuss further.

We have added text in several places to address these points:

Original text:
Further, the SSII is currently not well suited for rapidly expanding portions of species' ranges, such as in new invasions, which could be addressed through timely updates of range expectations or other invasion-specific information (McGeoch & Jetz 2019).

Updated text:
Further, because the SSII is currently based on static representations of species ranges, it does not capture range dynamics, such as in new invasions or range shifts (Lenoir et al. 2020). This could be addressed through timely updates of range expectations or other invasion-specific information (McGeoch & Jetz 2019). Dynamically tracking species distributions will be particularly important in cases where species ranges shift across national boundaries, resulting in new monitoring responsibilities.

Original text:
We consider expected diversity of terrestrial vertebrates to be largely static over our study period (1950-2019) with any species range shifts having little impact on the baseline broad-scale, cross-taxon diversity patterns while acknowledging that range expectations likely have higher uncertainty in the earliest decades.

Updated text:
Our estimates of expected terrestrial vertebrate diversity are based on static estimates of species ranges and thus does not capture range shifts which may have occurred during our study period (1950-2019) (Chen et al., 2011; J. Lenoir & Svenning, 2015; Jonathan Lenoir et al., 2020; Parmesan & Yohe, 2003; Pecl et al., 2017). Our use of species distributions coarsened to 110 km grid cells to reduce false presences, may help alleviate this issue, as the magnitude of shifts for terrestrial vertebrates will often fall within this expectation (Jonathan Lenoir et al., 2020). However, we acknowledge that species range shifts may impact even our broad-scale baseline of cross-taxon diversity patterns. Our data coverage metrics may be most strongly affected by recent range shifts driven by climate change (Jonathan Lenoir et al., 2020) as well as land-use change and interacting effects on species ranges (Guo et al., 2018; Sirami et al., 2017). However, the indices and associated analytical framework we have developed can flexibly support dynamic range expectation information as they become available.

Finally, as a relatively more minor concern, I think the authors should also better acknowledge the recent scientific literature highlighting the strong geographic, taxonomic but also methodological biases in species distribution and redistribution (as climate warms) which altogether suggests that no global biodiversity dataset or meta-analysis on biodiversity changes so far is truly global as it is most often claimed (see Brown et al. 2016; Feeley et al. 2017; Lenoir et al. 2020; Nunez & Amano 2021). See my specific comments below for more detailed suggestions which I hope the authors will find useful for their work.

Thank you for these suggestions, we have added these references throughout the manuscript.

Specific comments to the authors

Line 1: In the title, I think it is important to specify that this work focuses on terrestrial vertebrates only. Indeed, and although the same approach could be applied to other taxonomic groups as you mentioned in the text, this approach strongly relies on expert knowledge for species range maps, which is a strong limitation for a lot of very important taxonomic groups like plants or insects.

As the other reviewers attest, the development and introduction of the metrics represents a key contribution and novelty of the paper on which a specific empirical demonstration is then based. As we show in the revision, other input besides expert maps can serve to support metric calculations. We thus feel the title of the paper appropriately reflects the goal of the study , but we are of course happy to follow any preference from the Editors here.

Line 26: The sharp decline in sampling effectiveness is mathematically expected as SSII increases (see my general comments).

Please see our response to the related, general comment above.

Lines 34-35: Here, when mentioning the manifold consequences of biodiversity changes, you could cite some references like Pecl et al. (2017) or Bonebrake et al. (2018).

Thank you, we have added these references.

Lines 43-44: Indeed and databases on species range shifts already exist, like the BioShifts database (see Lenoir et al. 2020) which is freely available (https://doi.org/10.6084/m9.figshare.7413365.v1).

Thank you, we have added this reference.

Line 50: What about the need for long-term time series of monitoring data in order to assess biodiversity changes (see the BioTiME database from Dornelas et al. 2018)?

We have added references to the need for documentation of biodiversity change over time and the associated reference to the BioTiME database.

Original text:
Ambitions to limit threats to species and ensure the integrity of ecosystems, which are central goals of the post-2020 Global Biodiversity Framework under discussion (Convention on Biological Diversity 2020b), critically rely on effective documentation and monitoring of species distributions (Rounsevell et al. 2020; Visconti et al 2019).

Updated text:
Ambitions to limit threats to species and ensure the integrity of ecosystems, which are central goals of the post-2020 Global Biodiversity Framework under discussion (Convention on Biological Diversity 2020b), critically rely on effective documentation and monitoring of species distributions and changes over time (Dornelas et al. 2018; Rounsevell et al. 2020; Visconti et al 2019).

Lines 60-64: About taxonomic and geographic biases, the exact same pattern applies for data on species redistribution (see Feeley et al. 2017; Lenoir & Svenning 2015; Lenoir et al. 2020). Besides these biases, there is also important methodological biases in the way data are recorded and then used in subsequent quantitative analyses (see Brown et al. 2016; Lenoir et al. 2020). I think it is also worth pointing at in the list of biases that are currently acknowledged in the scientific literature.

We have added these references.

Line 65: Not only socio-economic and ecological drivers to explain data gaps but also linguistic drivers. Indeed languages have also been highlighted as important barriers for global science in general (see Amano et al. 2016).

We have updated the text and included the associated reference to incorporate linguistic barriers.

Original text: Scientists have identified a range of socio-economic and ecological drivers for gaps….

Updated text: Scientists have identified a range of socio-economic, linguistic, and ecological drivers for gaps….

Line 75: About growing data for plant species distribution at the global extent, are you aware of the sPlot database (Bruelheide et al. 2019)? This one also suffers from the same geographic biases.

We have added reference to the sPlot database in the discussion pertaining to mapping efforts for other taxa.

Line 85: See my general comment about the SSEI metric.

See response to general comment above.

Line 92: About the optimal spatial resolution of your grid (110 km * 110 km at the equator), how did you set it exactly? Why do you consider this spatial resolution the most appropriate and finest spatial resolution you can use? Why not trying a finer spatial resolution? Did you run a sensitivity analysis varying the size of the spatial resolution? This was not clear in the methods section (cf. lines 569-570).

We have provided empirical examples of how the SSII can be extended to finer spatial resolutions for two example species (Supplementary Fig. 3). We have added text throughout the manuscript to clarify the use of the current spatial resolution and implications for extending to finer resolutions.

Additional text (Main text):
Our methodology and analysis infrastructure is capable of flexibly accommodating different spatial resolutions (Supplementary Fig. 3, Supplementary Text) as more accurate information on species' ranges becomes available (i.e. through species distribution modeling) for a broader range of taxa.

Original text (Methods, Species distribution data):
Spatial biodiversity data coverage, hereafter referred to as data coverage, can be calculated at any spatial, temporal, or taxonomic resolution of interest. Doing so requires an estimation of expected biodiversity. We estimated terrestrial vertebrate diversity based on composites of single species distribution maps, which have been shown to approximate species' global extents over long time periods (ca. 20-40 years) (Gaston & Fuller, 2009; Hurlbert & Jetz, 2007) with minimum false presence rates at spatial grains of 100-500 km (Hurlbert & Jetz, 2007).

Updated text (Methods, Species distribution data):
Spatial biodiversity data coverage, hereafter referred to as data coverage, can be calculated at any spatial, temporal, or taxonomic resolution of interest. Doing so requires an estimation of expected biodiversity. The spatial resolution of analysis is primarily determined by the data used to estimate a species expected range. That is, higher spatial uncertainty in the species range expectations necessitates coarser resolutions of analysis in order to minimize false presences and thereby not hold nations accountable for collecting data in areas where a species does not occur. We estimated terrestrial vertebrate diversity based composites of single species distribution maps. Previous empirical studies have shown that such expert-based range maps minimize false presences at spatial resolutions of 100-500 km to approximate species' global extents over long time periods (ca. 10-50 years) (Gaston & Fuller, 2009; Hurlbert & Jetz, 2007; Jetz et al. 2012). Therefore, we estimated diversity using an equal-area grid with the finest spatial grain appropriate for expert-based species distribution maps (110km x 110km).

We demonstrated the potential to estimate species distributions at finer spatial resolutions (55 and 27.5 km) based on output from published species distribution models (Ellis-Soto et al. 2021) for two example species (Supplemental Fig. 3).

Additional text (Methods, Species Status Information Index (SSII):
We demonstrated the sensitivity of the SSII to a range of spatial resolutions (110, 55, and 27.5 km) for two example species based on published species distribution models (Ellis-Soto et al. 2021) and found that SSII values decrease predictably at finer spatial resolutions (Supplementary Fig. 3, Supplementary Text).

Supplementary results text (figure below):
We demonstrated the potential of the SSII to be calculated across a range of spatial resolutions (110, 55, and 27.5 km) for two example species (Supplemental Fig. 3). Because expert-based species distribution maps are not accurate at finer spatial resolutions, we used thresholded output from published species distribution models to estimate ranges at finer resolutions for two hummingbird species, the Glowing puffleg (*Eriocnemis vestita*) and White-sided hillstar (*Oreotrochilus leucopleurus*) (Ellis-Soto et al. 2021). Thresholded species distribution model outputs were rescaled to three equal-area grids (110, 55, and 27.5 km) and intersected with records collected over the previous two decades (2000-2019) (Supplemental Fig. 3 a,b). We computed annual SSII values over the same time period for both species. Unsurprisingly, data coverage decreased at finer spatial resolutions (Supplemental Fig. 3c). We compared SSII values based on each spatial resolution and estimated the slope of relationship for both species independently using linear regression (Supplemental Fig. 3d). For each comparison, we found that 95% confidence intervals of regression slopes overlapped between species, suggesting that SSII values scale consistently between resolutions among species. Therefore, while spatial scale clearly impacts the absolute value of the SSII, comparisons across species and regions are consistent across spatial resolutions.

Lines 97-98: What about trans-boundary shifts under contemporary climate change? Some species are shifting poleward in latitude and upward in elevation, thus changing and potentially reshuffling the national stewardships you used in your analysis. This is an important matter (see my general comments), no?

See response to general comment above.

Lines 101-102: Why constraining the SSEI metric to the realized spatial distribution of records? This is a bit misleading as you do not account for absence data in the other grid cells that are expected to be occupied. Yet, it is what matters in the end to get a uniform spatial distribution of records across all grid cells that are expected to be occupied, right? Why not penalizing the SSEI metric by the total number of grids cells supposed to be occupied but empty? Is it because you suppose that a grid cell expected to be occupied but empty could mean that the species is truly absent? To distinguish between that case (a true absence) and the other case of a false absence due to less sampling effort, you could use information from other species occurrence records during the same time period. I mean, if there is a high density of occurrence records for other terrestrial vertebrates in a grid cell where the focal species is absent but supposed to be occurring, then it is
likely to be a true absence, right?

Please see our response to the related general comment above.

Lines 112-113: Indeed, but this is expected since the more occurrence records you sample, the more likely it is to be unevenly distributed throughout a relatively larger number of occupied grid cells (higher SSII values).

Please see our response to the related general comment above.

Lines 121: Indeed, see also the recent release of: sPlot, the global vegetation plot database (Bruelheide et al. 2019) providing data on plant co-occurrence; BioTIME, the global database on biodiversity time series (Dornelas et al. 2018); or BioShifts, a database on species range shifts (Lenoir et al. 2020).

We have added these references on L54.

Updated text:
Thanks to significant advances in data collection, mobilization, and aggregation, publicly accessible occurrence data are growing rapidly (Dornelas et al. 2018, Bruelheide et al. 2019, Lenoir et al. 2020), with over 1.6 billion occurrence records across sources and taxa available in the Global Biodiversity Information Facility (GBIF).

Lines 128-129: Again, this is very much expected and not surprising given how SSEI works (see my general comments).

Please see our response to the related general comment above.

Line 135: What do you mean exactly by "slowed in their coverage progress"? Do you mean according to SSII or according to SSEI? Or both?

We have added text clarifying that this refers to the SSII.

Lines 150-153: According to the realized distribution yes, it is true that sampling effectiveness has decreased in countries with increased SSII (expected pattern), but according to the expected distribution, this is not necessarily the case, right? Again, I think the way the SSEI is constructed is problematic as it is specifically focused on the realized distribution of records and it is completely blind to the expected distribution of records (cf. it does not account for the distribution of cells expected to be occupied but empty).

Please see our response to the related general comment above.

Lines 165-170: This pattern for birds (increasing SSII but decreasing SSEI) is likely due to the fact that citizen science data are everything except strategic sampling and thus the increase in spatial coverage in occurrence records automatically comes with a decrease in the spatial evenness of those records across the sampled area, this is well expected (see my general comments). The more data we collect, the more it is likely to be unevenly distributed in space

because humans never collect data randomly in the field. Once a good spatial coverage in occurrence records is achieved, one needs to invest in strategic sampling, which cannot really be achieved by opportunistic data, except under strong guidance by the scientific community to orient citizen science data towards a strategic sampling. I think this is something important to discuss. The rapid increase in citizen science data in ecology is a great thing but it is definitely not the most efficient approach to reach a strategic sampling design if citizen science is not undertaken under the guidance of the scientific community. Not even mentioning the issue of data quality, this type of opportunistic and chaotic data (in terms of spatial distribution) comes with costs as it is then very difficult to use and analyse such data when monitoring biodiversity changes over time. Opportunistic data as collected by GBIF cannot really replace a strategic sampling that is designed for the purpose to assess biodiversity changes. Maybe this could be discussed and highlighted to better balance the discussion around citizen science data.

We certainly agree with this sentiment and have added the following text to support this.
Original text:
The accelerated pace of data coverage for birds compared to other vertebrate groups points to the tremendous role that non-museum based data collection can play in closing knowledge gaps (Pocock et al., 2018, 2019; Theobald et al., 2015). Expanding the impact of citizen science initiatives for information growth will likely benefit from initiatives and guidance addressing the most effective and complementary contributions.

Updated text:
However, the rapid decline in sampling effectiveness we found for bird species, coincident with the growth in citizen science platforms, suggest that this data has not collected to optimally support closing knowledge gaps. While the contributions of citizen science have been invaluable, eExpanding the impact of citizen science initiatives for information growth will likely benefit from initiatives and guidance addressing the most effective and complementary contributions (i.e. addresses undersampled species or regions)

Lines 172-174: Ok, but this comes with costs (see my comment just above) and it should be discussed. I have the impression that the discussion is only oriented towards the benefits of citizen science data without discussing its drawbacks (e.g. uneven distribution of data).

We agree with the reviewer and our intention was to highlight these issues through the following statements, in addition to the statement added in response to the comment above.

"However, this onslaught of observations has not been maximally leveraged to enhance the global biodiversity information base, as seen in the coincident decline in avian sampling effectiveness (Fig. 3e)....
However, the rapid decline in sampling effectiveness we found for bird species, coincident with the growth in citizen science platforms, suggest that this data has not collected to optimally support closing knowledge gaps. While the contributions of citizen science have been invaluable, eExpanding the impact of citizen science initiatives for information growth will likely benefit from initiatives and guidance addressing the most effective and complementary contributions (i.e. addresses undersampled species or regions)."

Lines 176-178: Indeed, this is very important and you could expand a bit this part of the discussion (see my comments above).

We have added the following statement to further support this point.

Additional text:
Citizen science platforms could shift incentives from numbers of records collected or species identified to the value of records contributed.

Lines 183-184: Linguistic factors as well (Amano et al. 2016).

We have added this reference.

Lines 184-188: Why did you only use the SSII metric when building your categories? I find it a bit strange that you did not also incorporate the SSEI metric in your categorization. Maybe this is somewhat related to my comment on the fact that SSEI is a bit meaningless under low values of SSII.

Our goal here was to discuss status and trends in data coverage and highlight various typologies (i.e. low, but increasing coverage vs. high, but stagnating coverage). We envision the SSEI as supporting decision making on how to best increase the SSII (i.e. but reallocated effort, resources from places and taxa with a high degree of redundancy).

Lines 210-212: Can you provide examples of countries reflecting this situation?

We have added national examples.

Lines 214-225: Here, you discuss the drawbacks of the SSII metric, which is nice, but what about the drawbacks of the SSEI metric (cf. my general comments on the issue that SSEI is not really informative for low values of SSII)? Besides, when discussing the issue of invasive species for the SSII metric, you should also remind the reader about the static nature of SSII (cf. you do not account for potential species range shift under climate change) and the fact that trans-boundary shifts of native species under anthropogenic climate change may completely change the pattern you found for SSII, and especially so for steward's SSII.

We have added discussion of these issues, see response to general comments above.

Line 235: Species-level expectations should account for species redistribution under contemporary climate change, this is not a minor issue (see my general comments).

Line 238: Please provide citations to illustrate this increase in mapping efforts for other taxonomic groups and systems such as plants (see Bruelheide et al. 2019) and marine taxa (cf. OBIS data).

Thank you, we have added references to this statement.

Lines 322-323: Shading are not visible in panel c. Is it because of very low 95%CI?

Yes, this is due to a very low 95% confidence interval.

I sincerely hope that my comments and suggestions will help both the authors to improve their work as well as the editorial board to take the right decision on the present manuscript. It was a real pleasure to read and review this inspiring work.

Jonathan Lenoir

References

Amano et al. (2016) Languages Are Still a Major Barrier to Global Science. PLoS Biology, 2000933

Bonebrake et al. (2018) Managing consequences of climate-driven species redistribution requires integration of ecology, conservation and social science. Biological Reviews, 93, 284-305

Brown et al. (2016) Ecological and methodological drivers of species' distribution and phenology responses to climate change. Global Change Biology, 22, 1548-1560

Bruelheide et al. (2019) sPlot - A new tool for global vegetation analyses. Journal of Vegetation Science, 30, 161-186

Chen et al. (2011) Rapid range shifts of species associated with high levels of climate warming. Science, 333, 1024-1026

Dornelas et al. (2018) BioTIME: A database of biodiversity time series for the Anthropocene. Global Ecology and Biogeography, 27, 760-786

Feeley et al. (2017) Most "global" reviews of species' responses to climate change are not truly global. Diversity & Distribution, 23, 231-234

Lenoir & Svenning (2015) Climate-related range shifts - a global multidimensional synthesis and new research directions. Ecography, 38, 15-28

Lenoir et al. (2020) Species better track climate warming in the oceans than on land. Nature Ecology & Evolution, 4, 1044-1059

Parmesan et al. (2003) A globally coherent fingerprint of climate change impacts across natural systems. Nature, 421, 37-42

Pecl et al. (2017) Biodiversity redistribution under climate change: Impacts on ecosystems and human well-being. Science, 355, eaai9214
