# Supplementary material for: Global and national trends, gaps, and opportunities in documenting and monitoring species distributions
Source: PLoS Biol. 2021 Aug 12;19(8):e3001336. doi: 10.1371/journal.pbio.3001336 (PMC8360587; doi:10.1371/journal.pbio.3001336)
Supplement: Attachment — Submitted filename: Oliver_etal_SSII_reviews_6-17-2021.pdf [file pbio.3001336.s003.pdf]

Dear Dr. Roberts,

We sincerely thank you, the Associate Editor, and reviewers for your additional time and feedback. We feel the reviewers' suggestions have greatly improved the manuscript. Below, please find our detailed responses to each of the reviewers' comments. We have updated the sensitivity analysis to include the SSEI and subsequently updated Supplementary Figure 3. We have included text clarifying the connection of the SSEI to previous metrics, as suggested. We have also updated the title. In addition, we have provided all data and scripts to support the manuscript on a public GitHub repository. We have also provided public download of national indicator values from the Map of Life website to further support exploration and tracking of indicators as future updates become available. We thank you for your further consideration of our work.

Best,
Ruth Oliver

IMPORTANT:

a) Your findings are somewhat nuanced, but we wondering whether they could be indicated in some way in your Title to make it more informative (something like "Global and national trends in documenting and monitoring species distributions identify opportunities to close critical information gaps").

We appreciate the suggestion to incorporate opportunities to close information gaps into the tile. We kindly suggest the following to address this.

Global and national trends, gaps, and opportunities in documenting and monitoring species distributions

b) Please attend to the remaining requests from revs #3 and #4. The Academic Editor particularly wanted me to stress the need to ensure that the precedence for SSEI is made clear, as requested by rev #4 ("I would like to see the authors acknowledge the equivalence of SSEI with preceding indices (Lenoir review) and tone down claims of novelty. The results are important without this claim.").

Thank you, we have responded to this comment in the response to Reviewer #4 and have added language to clarify the connection to the other approaches highlighted.

c) You'll see that rev #1 raises strong concerns about data availability (as did rev #3 in the previous round). This is clearly an important and emotive issue in the community, and we encourage you to do your utmost on this front. Specifically, we remind you of your commitment previously expressed in the Response to Reviewers: "MOL has submitted an application to be formally recognized as a trusted data repository. If that status is not in place by the time of publication, all information necessary to replicate the results will in addition to on MOL also be made available on a formally already recognized repository."

We have provided all data and code to support the replication of our results and figures via a public GitHub repository with an associated DOI. We have also provided the national indicator values for direct, public download from MOL. We are excited to host the national data on the MOL site, so that the public can continue to track these indicators as they are updated in the future. We are happy to provide any additional information or alternative formats.

All data to replicate results are present in the GitHub repository.
https://github.com/MapofLife/biodiversity-data-gaps
https://doi.org/10.48600/MOL-3Y3Z-DW77
Data is also directly downloadable from MOL at mol.org/indicators/coverage.

d) You should also familiarise yourself with our data policy. This does have exemptions for third party data, but please could you provide information on the license or a letter/email from the third party (IUCN), explicitly stating that the data cannot be shared even with appropriate credits? If the data can be shared, then you should do so.

e) Please attend to my Data Policy requests below. Specifically, we will require any code needed to reproduce your results to be made available as supplementary files or in e.g. Github. We'll also need the numerical values underlying Figs 2CDEFG, 3ABCDEF, 4CF, 5ABC, S1ABCD, S2AB, S3CDEF. In addition, please cite the location of the data clearly in each relevant Fig legend.

We have provided all data and code to reproduce the figures via a public GitHub repository with an associated DOI.
https://github.com/MapofLife/biodiversity-data-gaps
https://doi.org/10.48600/MOL-3Y3Z-DW77

As you address these items, please take this last chance to review your reference list to ensure that it is complete and correct. If you have cited papers that have been retracted, please include the rationale for doing so in the manuscript text, or remove these references and replace them with relevant current references. Any changes to the reference list should be mentioned in the cover letter that accompanies your revised manuscript.

We expect to receive your revised manuscript within two weeks.

To submit your revision, please go to https://www.editorialmanager.com/pbiology/ and log in as an Author. Click the link labelled 'Submissions Needing Revision' to find your submission record. Your revised submission must include the following:
- a cover letter that should detail your responses to any editorial requests, if applicable, and whether changes have been made to the reference list
- a Response to Reviewers file that provides a detailed response to the reviewers' comments (if applicable)
- a track-changes file indicating any changes that you have made to the manuscript.

NOTE: If Supporting Information files are included with your article, note that these are not copyedited and will be published as they are submitted. Please ensure that these files are legible and of high quality (at least 300 dpi) in an easily accessible file format. For this reason, please be aware that any references listed in an SI file will not be indexed. For more information, see our Supporting Information guidelines:
https://journals.plos.org/plosbiology/s/supporting-information

Published Peer Review History
Please note that you may have the opportunity to make the peer review history publicly available. The record will include editor decision letters (with reviews) and your responses to reviewer comments. If eligible, we will contact you to opt in or out. Please see here for more details:

https://blogs.plos.org/plos/2019/05/plos-journals-now-open-for-published-peer-review/

Early Version
Please note that an uncorrected proof of your manuscript will be published online ahead of the final version, unless you opted out when submitting your manuscript. If, for any reason, you do not want an earlier version of your manuscript published online, uncheck the box. Should you, your institution's press office or the journal office choose to press release your paper, you will automatically be opted out of early publication. We ask that you notify us as soon as possible if you or your institution is planning to press release the article.

Protocols deposition
To enhance the reproducibility of your results, we recommend that if applicable you deposit your laboratory protocols in protocols.io, where a protocol can be assigned its own identifier (DOI) such that it can be cited independently in the future. Additionally, PLOS ONE offers an option for publishing peer-reviewed Lab Protocol articles, which describe protocols hosted on protocols.io. Read more information on sharing protocols at https://plos.org/protocols?utm_medium=editorial-email&utm_source=authorletters&utm_campaign=protocols

Please do not hesitate to contact me should you have any questions.

Sincerely,

Roli Roberts
DATA POLICY:

You may be aware of the PLOS Data Policy, which requires that all data be made available without restriction: http://journals.plos.org/plosbiology/s/data-availability. For more information, please also see this editorial: http://dx.doi.org/10.1371/journal.pbio.1001797

Please make all code required to reproduce your results available, either as a supplementary file or in a repository such as Github. We also require all the numerical values underlying the figures and results of your paper be made available in one of the following forms:

1) Supplementary files (e.g., excel). Please ensure that all data files are uploaded as 'Supporting Information' and are invariably referred to (in the manuscript, figure legends, and the Description field when uploading your files) using the following format verbatim: S1 Data, S2 Data, etc. Multiple panels of a single or even several figures can be included as multiple sheets in one excel file that is saved using exactly the following convention: S1_Data.xlsx (using an underscore).

2) Deposition in a publicly available repository. Please also provide the accession code or a reviewer link so that we may view your data before publication.

Regardless of the method selected, please ensure that you provide the individual numerical values that underlie the summary data displayed in the following figure panels as they are essential for readers to assess your analysis and to reproduce it: Figs 2CDEFG, 3ABCDEF, 4CF, 5ABC, S1ABCD, S2AB, S3CDEF. NOTE: the numerical data provided should include all replicates AND the way in which the plotted mean and errors were derived (it should not present only the mean/average values).

IMPORTANT: Please also ensure that figure legends in your manuscript include information on where the underlying data can be found, and ensure your supplemental data file/s has a legend.

Please ensure that your Data Statement in the submission system accurately describes where your data can be found.

DATA NOT SHOWN?

- Please note that per journal policy, we do not allow the mention of "data not shown", "personal communication", "manuscript in preparation" or other references to data that is not publicly available or contained within this manuscript. Please either remove mention of these data or provide figures presenting the results and the data underlying the figure(s).

REVIEWERS' COMMENTS:

Reviewer #1:

I sincerely appreciate the work put forth by the authors here. After seeing the decision come through, I thought "wow - four massive reviews - that is a lot of work". I'm glad, however, that the authors decided to undertake this work. I believe they struck a nice balance of appeasing Reviewers and also strengthening their manuscript, without going down too many rabbit holes. Well done.

All in all, I believe the text is sufficiently revised for publication.

BUT, I have one sticking point. And ultimately, this is up to the Editorial Board at PLoS Biology. This was also highlighted by Reviewer 3 in the original round of review. That is 'data availability'.

I mean no offense, but I strongly disagree with the notion of "visually accessible" (the wording in the current data availability statement). To be abrupt, I don't think I've seen this wording yet. And I honestly don't know what this means, but to me, it does not meet the notion of data accessibility/availability that we as a conservation biology community should be striving for. And even for the indicators information, I still don't know how to get these data. I went to https://mol.org/indicators/ as suggested by the data availability statement. But I still don't see any 'download' button or any way to get the data. All I see is 'explore'. I believe that an 'account' should not need to be created in order to access these data (I'm partially assuming that is how I can download the entire dataset). These data need to be made available with the paper as a current record of downloadable and reproducible research in my opinion. All the data, not simply one country at a time. Especially, for such a prestigious outlet of PLoS Biology. I understand the idea of IUCN not making range data available (which is a sticky problem in and of itself) and your hands being tied, so to speak. But regardless, the authors should strive to make as much data available as possible to reproduce these results. I apologize if this sounds harsh, but I think if real change is going to ever happen in our (the scientific community, specifically ecology/biodiversity research) push for available data and reproducibility then it starts with big/prestigious labs (e.g., Walter's group) publishing papers in big outlets (e.g., PLoS Biology) going the extra mile to ensure the data are available and results reproducible beyond "visually accessible".

Best of luck with your future work and I look forward to seeing this manuscript online soon!

We sincerely apologize for any misunderstanding. Our intention was to indicate that if MOL's formal repository status was not obtained by the time publication we would happily provide all necessary data in the desired format. We have provided all supporting data via a GitHub repository where all data and code to support the manuscript can be found with an associated DOI. We have also provided the national indicator data for direct, public download on MOL to support updates in the future and long-term tracking of these metrics.

https://github.com/MapofLife/biodiversity-data-gaps
https://doi.org/10.48600/MOL-3Y3Z-DW77
mol.org/indicators/coverage

Reviewer #3:
[identifies himself as Dominique Roche]

The authors have put considerable work into addressing the reviewer comments and . The only comment I have pertains to the need to present test statistics (and effect sizes, when possible) alongside p values in the main text and the ESM. P values should also be reported to three decimal places. Although not necessary, it would be helpful if the authors also included the sample size or degrees of freedom as appropriate. I cannot indicate line numbers as there are none in the manuscript.

Thank you for the feedback. We have attempted to address these issues to improve clarity on the test statistics we are reporting. Several of the examples highlighted pertain to the assessment of national trends in SSII/SSEI. For this portion of the analysis we are reporting the proportion of nations for which significant trends were detected, as assessed at the $p < 0.01$ threshold. Therefore the effect sizes etc. vary by individual countries. Full test statistics for each nation can be found in the supporting data and code.

Examples include:

Main text:

"Globally, only approximately half of nations (42%) showed increasing, significant trends ($p < 0.01$) in coverage averaged across taxa over the previous decade (Fig. 4b)."

See comment above.

Captions for Figs 4 and 5. The term 'significant' is used repeatedly without reference to the ESM where the methods and statistical results are presented.

Added references to significance level in caption.

ESM:

"The temporal patterns in SSII are different, with birds only exceeding the three other groups after 1980, but since then showing near linear-growth in taxon-wide SSII and exceeding other classes in 2019 by nearly 10-fold (Fig. 3c)." What statistics were used to infer these results? If it was a comparison of confidence intervals, perhaps re-state here for clarity.

We are simply referencing the fact that SSII for bird species was greater than that for other taxa based on the mean in the period after 1980. In the associated figure we show the mean with a 95% confidence interval, as described in the figure caption. We have added in text to clarify that this statement is referencing the mean.

Updated text:
The temporal patterns in mean SSII are different, with birds only exceeding the three other groups after 1980, but since then showing near linear-growth in taxon-wide mean SSII and exceeding other classes in 2019 by nearly 10-fold (Fig. 3c).

"Steward's SSII has recently increased in a majority of nations (84%), particularly in North America and southern and eastern Europe with nearly half of nations (42%) showing significant ($p < 0.001$) increasing trends (Fig. 3b). Of the minority (13%) with decreasing rates, Finland had the most rapid decrease (-0.021 SSII/year). Despite mostly positive trends, much of Africa and Asia saw only negligible increases in indicator values over the last decade, with the exceptions of India, Sri Lanka, and South Korea which showed large increases in data coverage. Nations were nearly evenly split between either non-significant and significantly increasing Steward's SSII for resident bird species (52.8% and 47.2%, respectively, none decreasing; Fig. 3c). Most nations did not have significant trends in data coverage for mammals (85.8%), amphibians (89.9%), and reptiles (81%)."

See comment above.

"Recent National SSEI differed strongly among nations (Fig. 4d, Supplementary Table 3). National SSEI was generally lower within western Europe, North America, and Australia. National SSEI and Steward¶s SSII were weakl\, negativel\ correlated (Spearman¶s rho = -0.52, $p < 0.01$). A majority of nations (51%) had decreasing SSEI across terrestrial vertebrates, however only 11% of nations globally had significant ($p < 0.01$), decreasing trends (Fig. 3e). These nations included the United States, Canada, Italy, and South Africa. Decreasing trends in SSEI were most common for bird species (27.5%) (Fig. 4f)."

Etc.

See comment above.

Reviewer #4:
[identifies himself as Jonathan Lenoir]

General comments

I was one of the four reviewers (reviewer #4) during the former round of review. I read the authors' responses to my comments (as well as their responses to the comments from the other three reviewers) and I particularly appreciate the efforts made by the authors to address most of the concerns collectively arising from the four reviewers. For instance, the new Supplementary Fig. 3 on the impact of varying grain sizes on the SSII metric is a great addition in terms of sensitivity analysis, as requested by reviewers #1 and #3. But then, why not also assessing the impact of varying spatial grains on the other metric: SSEI?

Or is it because the SSEI metric is unaffected by the variation in the spatial grain? If so, it would be nice to precise it and show it.

We have added the same spatial sensitivity assessment for SSEI in Supplementary Figure 3.

About SSEI, I am also very grateful to the authors for considering my main initial concern on the potential link between SSII and SSEI. The additional explanations provided by the authors and the new panel in Supplementary Fig. 4 are really helpful in that respect. Also, the important clarification on the special case of just a single grid cell where there is one or several records of occurrence for the focal species is indeed important. In fact, such cases would lead to a maximum entropy (H*) being equal to zero (log(1)=0) and thus the SSEI index would equal Inf value. So, I am grateful to the authors for also clarifying this issue.

Thank you, we are glad this clarified the issue and feel it strengthened the manuscript overall.

This said, I am still convinced that the SSEI metric is only useful when N, the total number of records (i.e. total number of occurrence records for a given species), is several order of magnitude larger (e.g. 10 to 100 times at least) than G, the total number of grid cells where there is actual information on sampling effort. Indeed, both panels (a) and (b) in Supplementary Fig. 4 are actually illustrating this very well since SSEI can only reach very low values and thus be highly variable (which is a very important feature for discriminating different sampling effectiveness situations) when the total number of records is way larger than the total number of grid cells sampled. So, I would at least recommend to discuss that inherent property of the SSEI metric and recommend the authors to warn the reader about this and that the SSEI metric will be especially relevant under high sampling effort (in terms of total number of occurrence records) relative to the number of grid
cells that are sampled.

We have added the following text to the Methods section to clarify this point.

Additional text:
As SSEI is based on normalized entropy, its value does not depend directly on total sampling effort (i.e. total number of records, N), though we note that the range and resolution of the SSEI does depend on N. Thus while SSEI can be compared between species with different overall sampling effort, some care should be taken in interpreting SSEI when sampling effort varies by orders of magnitude or when sampling effort is exceptionally low.

Still about the SSEI metric and information theory (IT), I would like to mention here that this metric is, simply put, the Pielou's index of diversity, also called equitability or evenness index (cf. the empirical entropy measured by Shannon's index divided by the maximum entropy given by Shannon's index). The analogy with the way the Shannon's index is used in Ecology to measure species richness is that maximum entropy is given by H*=log(S) where S is the total number of species and then N is the total number of individuals, with ni being the number of individuals for species i. Here, the authors did not consider S but G, the total number of grid cells sampled with N being the total number of occurrence records and not the total number of individuals. Hence, there is nothing new or novel for me with the SSEI index itself because it is a metric that already exists in IT and that is widely used in ecology. Hence, the authors' claim in the abstract that "we propose novel indicators of biodiversity data coverage and sampling effectiveness" is an overstatement to me. Yet, the idea of the authors to borrow the Shannon's and Pielou's index from IT for assessing the equitability of the sampling of occurrence records over the grid cells where the species is known to occur is indeed a novel application of the Pielou's index and an interesting one. This is why I think this study has definitely a great potential, not because of the SSEI

metric itself (nothing new with that because it is Pielou's index) but because of its application to assess sampling effectiveness across the set of grid cells where data is available. So, maybe it would be nice that the authors tone down this claim of "novelty" on the SSEI metric and actually refer to the Pielou's index when mentioning the SSEI metric, just to relate to existing metrics from IT

We have added the following text to acknowledge the connection to Pielou's index.

Additional text:
The SSEI is similar to other information theoretic evenness metrics, such as Pielou’s index of species evenness which is also based on normalized entropy (Pielou, 1966).

By the way, I actually think there is a mistake in the text explaining the SSEI metric as the formula written in the Supplementary Materials, Methods section (SSEI subsection), for H* (cf. H*=log(N)) is wrong. Indeed, N is the total number of records here, while the formula of maximum entropy should be H*=log(G), where G is the total number of sampled grid cells. I assume that only the formula of H* in the text of the Supplementary Materials is wrong and that the authors actually used the right formula in their computations and analyses, but it would be nice that the authors confirm this is actually the case. Note that the authors are sometimes mixing G with N in their responses to my former comments. For instance, when mentioning the special case of a single grid cell (G), the authors mentioned a single occurrence record (N), which is different. Indeed, the same issue applies with several occurrence records if they all fall inside the same grid cell Gi, so it is the total number of sampled grid cell G that matters here and which should be strictly greater than 1.

Thank you for identifying this typo, we have corrected it in the text. As suggested, this was not an issue in the calculations of SSEI.

Sorry to insist on the metrics used here but these are quite central to the whole study. In that respect, I invite the authors to also consider the Simpson's index for assessing the evenness and equitability of the sampling across the sampled grid cells (cf. SSEI). Indeed, the Simpson's index has the advantage to account for the total number of grid cells that are actually sampled which is not the case for the Pielou's index used by the authors. Indeed, under perfectly even sampling effort, Pielou's index will give the exact same value of 1, or perfect evenness, if just 2 or 1000 grid cells are sampled while the Simpson's index will give a higher value for the situation in which more grid cells are sampled.

About my second major concern regarding species range shifts over time, the authors did a good job to address this point and to acknowledge the existing scientific literature on the matter. I have nothing else to add at this stage regarding this second concern I had. I agree with the authors that it would be too much to integrate in this study the temporal dynamic of species range shifts but it is good that the authors discuss their approach in light of this and that it can be implemented in the future given the increasing amount of biodiversity time series.

Thank you, we appreciate the feedback.

Again, I would like to thank the authors for addressing and answering my initial comments and concerns. I hope these new suggestions in light of the revised version of their work will further help them.

Yours sincerely,

Jonathan Lenoir
